# Kinetic Study of the Oxidative Addition Reaction between Methyl Iodide and [Rh(imino-β-diketonato)(CO)(PPh)_3_] Complexes, Utilizing UV–Vis, IR Spectrophotometry, NMR Spectroscopy and DFT Calculations

**DOI:** 10.3390/molecules27061931

**Published:** 2022-03-16

**Authors:** Hendrik Ferreira, Marrigje Marianne Conradie, Jeanet Conradie

**Affiliations:** 1 Department of Chemistry, University of the Free State, Bloemfontein 9300, South Africa; hendrik1ferreira@gmail.com (H.F.); conradj@ufs.ac.za (J.C.); 2 Department of Chemistry, UiT The Arctic University of Norway, N-9037 Tromsø, Norway

**Keywords:** rhodium, imino-β-diketonato, oxidative addition, DFT

## Abstract

The oxidative addition of methyl iodide to [Rh(β-diketonato)(CO)(PPh)_3_] complexes, as modal catalysts of the first step during the Monsanto process, are well-studied. The β-diketonato ligand is a bidentate (BID) ligand that bonds, through two O donor atoms (O,O-BID ligand), to rhodium. Imino-β-diketones are similar to β-diketones, though the donor atoms are N and O, referred to as an N,O-BID ligand. In this study, the oxidative addition of methyl iodide to [Rh(imino-β-diketonato)(CO)(PPh)_3_] complexes, as observed on UV–Vis spectrophotometry, IR spectrophotometry and NMR spectrometry, are presented. Experimentally, one isomer of [Rh(CH_3_COCHCNPhCH_3_)(CO)(PPh_3_)] and two isomers of [Rh(CH_3_COCHCNHCH_3_)(CO)(PPh_3_)] are observed—in agreement with density functional theory (DFT) calculations. Experimentally the [Rh(CH_3_COCHCNPhCH_3_)(CO)(PPh_3_)] + CH_3_I reaction proceeds through one reaction step, with a rhodium(III)-alkyl as the final reaction product. However, the [Rh(CH_3_COCHCNHCH_3_)(CO)(PPh_3_)] + CH_3_I reaction proceeds through two reaction steps, with a rhodium(III)-acyl as the final reaction product. DFT calculations of all the possible reaction products and transition states agree with experimental findings. Due to the smaller electronegativity of N, compared to O, the oxidative addition reaction rate of CH_3_I to the two [Rh(imino-β-diketonato)(CO)(PPh)_3_] complexes of this study was 7–11 times faster than the oxidative addition reaction rate of CH_3_I to [Rh(CH_3_COCHCOCH_3_)(CO)(PPh_3_)].

## 1. Introduction

**Scheme 1:**
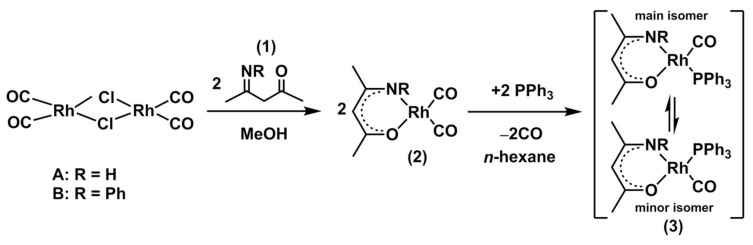
Synthesis of Rh complexes [Rh(CH_3_COCHCNHCH_3_)(CO)_2_] **2A** [Rh(CH_3_COCHCNPhCH_3_)(CO)_2_] **2B,** [Rh(CH_3_COCHCNHCH_3_)(CO)(PPh_3_)] **3A** and [Rh(CH_3_COCHCNPhCH_3_)(CO)(PPh_3_)] **3B** from (CH_3_COCH_2_CNHCH_3_) **1A** and (CH_3_COCH_2_CNPhCH_3_) **1B**.

Rhodium complexes are used in several industrial processes as catalysts [1,2,3]. Rhodium complexes, as catalysts, are used, for example, in the hydrosilylation crosslinking of silicone rubber [4], for making nitric acid [5] and in the hydrogenation of benzene to cyclohexane [6]. The [Rh(β-diketonato)(CO)(PPh)_3_] complex, with (β-diketonato = acetylacetono (acac)) as a hex-1-ene hydroformylation catalyst, produced 68% hex-2-ene [7]. Probably the most well-known example of a rhodium process used in the industry is the rhodium catalyzed carbonylation of methanol to produce acetic acid by the Monsanto process [8,9,10], where the first organometallic step involves the oxidative addition of methyl iodide to *cis*-[Rh(CO)_2_I_2_]^−^. [Rh(β-diketonato)(CO)(PPh)_3_] complexes, as model catalysts of the first step of the Monsanto process, are well-studied [11,12]. It is found that more electronegative groups attached to the backbone of the β-diketonato ligand and slowed down the oxidative addition reaction rate of methyl iodide to [Rh(β-diketonato)(CO)(PPh)_3_] complexes [11]. The β-diketonato ligand is a bidentate (BID) ligand that bonds through two O donor atoms (O,O-BID ligand) to rhodium. Imino-β-diketones are similar to β-diketones, though the donor atoms are N and O, hereby referred to as a N,O-BID ligand. The influence of the electronegativity of the two donor atoms (O,O) vs. (N,O) on the rate of oxidative addition of methyl iodide to the [Rh(L,L′-BID)(CO)(PPh)_3_] complexes, [Rh(CH_3_COCHCNHCH_3_)(CO)(PPh_3_)] **3A** and [Rh(CH_3_COCHCNPhCH_3_)(CO)(PPh_3_)] **3B** (Scheme 1), is presented in this research article. Results obtained by different experimental methods (UV–Vis spectrophotometry, IR spectrophotometry and NMR spectrometry) are presented. A density functional theory (DFT) study shed further light on experimental findings.

## 2. Results and Discussion

### 2.1. Synthesis of Rhodium(I) Complexes

The Rh-dicarbonyl complexes [Rh(CH_3_COCHCNHCH_3_)(CO)_2_] **2A** and [Rh(CH_3_COCHCNPhCH_3_)(CO)_2_] **2B** were synthesized from μ-dichloro-tetracarbonyldirhodium (obtained from Sigma Aldrich, Dramstadt, Germany) and the appropriate ligands, (CH_3_COCH_2_CNHCH_3_) **1A** and (CH_3_COCH_2_CNPhCH_3_) **1B**, respectively, in a methanol solution, see Scheme 1. The red methanol solution of the dirhodium complex turned to a yellow colour after the addition of the ligands. The extraction of the dicarbonyl complexes was performed with *n*-hexane, until the organic solvent that was used to extract it was clear. The collected solvent containing the product was then concentrated by evaporation. After a fine powder crystallized, the solution was filtered again, and the complex crystallized slowly from the *n*-hexane solution. Varshavsky reported the synthesis and properties of **2A** [13] and related Rh-dicarbonyl complexes containing β-aminivinyl ketones [14]. The structure of **2B** was confirmed by solid state crystal analysis [15].

Synthesizing the triphenylphosphine, containing Rh complexes [Rh(CH_3_COCHCNHCH_3_)(CO)(PPh_3_)] **3A** and [Rh(CH_3_COCHCNPhCH_3_)(CO)(PPh_3_)] **3B**, was performed similarly to the method of various researchers, when synthesizing Rh-monocarbonyl-triphenylphosphine-β-diketonato complexes from Rh-dicarbonyl-β-diketonato complexes [16,17,18]. The slow addition of the triphenylphosphine hexane solution to the dicarbonyl Rh complex hexane solution yielded tiny bubbles of CO gas, formed during the reaction. The reaction was left approximately 5 min, until no more bubbles were formed and the flask was then left at room temperature (with a slight draft in open air). The crystallization of the final products was performed in a manner similar to the dicarbonyl complexes. Varshavsky reported the synthesis and properties of many related Rh-monocarbonyl-β-ketoiminato complexes containing tertiary phosphines [19]. Two isomers are possible for each of **3A** and **3B**—one with the N_β-ketoiminato_ *trans* to CO (N-*trans*-CO) and one with O_β-ketoiminato_ *trans* to CO (O-*trans*-CO), as shown in Scheme 1. On the ^1^H-NMR 8.6% **3A** isomer, N-*trans*-CO and 91.4% **3A** isomer O-*trans*-CO is observed, while 100% **3B** isomer O-*trans*-CO and no **3B** isomer N-*trans*-CO is observed. The major isomer of **3A** and **3B**, observed as the O-*trans*-CO form, agrees with the fact that the **3A** isomer O-*trans*-CO [20] and molecules related to **3B** isomer O-*trans*-CO [21,22,23,24], could be isolated and characterized by solid state crystal structures.

On IR, it was not possible to distinguish between the different isomers of **3A** and **3B**—only one peak, corresponding to the CO stretching, was observed, at 1952.7 (**3A**) and 1966.6 (**3B**) cm^−1^. The IR stretching frequencies of the carbonyl group in **3A** and **3B** are at lower wavenumbers than that of the dicarbonyl complexes **2A** (2044.1 and 1971.0 cm^−1^) and **2B** (2059.0 and 1997.7 cm^−1^), respectively. This is indicative of a strengthening of the CO bonds, due to the addition of the more electron-rich triphenylphosphine ligand to the complex. The more electronegative groups or atoms will attract electrons in a bond more towards themselves, therefore slightly shortening and, thus, strengthening the bonds—which shifts the stretching frequency to smaller values.

### 2.2. DFT Study of Rhodium(I) and Rhodium(III) Complexes

#### 2.2.1. Rhodium(I)

**Figure 1:**
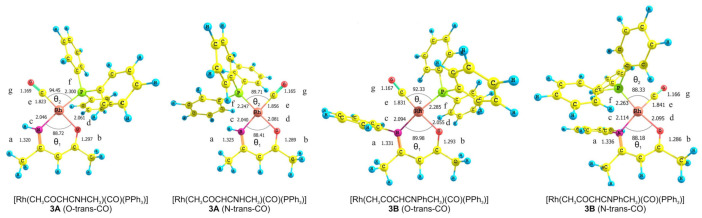
PW91/TZP DFT optimized structures of the possible isomers of the indicated [Rh(N,O-BID)(CO)(PPh_3_)] complexes with selected bond distances (in angstrom) and bonding angles (in degrees).

**Figure 2:**
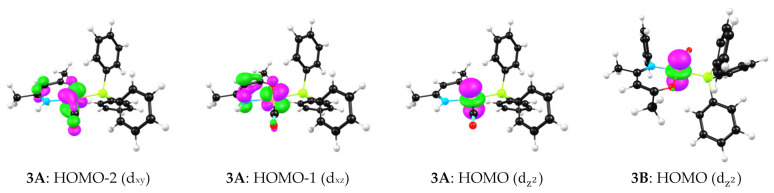
Selected Kohn-Sham frontier orbitals of the DFT PW91/TZP optimized geometry of the main isomers (O-*trans*-CO) of [Rh(CH_3_COCHCNHCH_3_)(CO)(PPh_3_)] **3A** and [Rh(CH_3_COCHCNPhCH_3_)(CO)(PPh_3_)] **3B**.

The density functional theory (DFT) calculated optimized structures of the two possible isomers each of **3A** and **3B** are shown in Figure 1. The complexes have a square planar geometry, though the **3B** isomer N-*trans*-CO exhibits a distorted square planar geometry—due to the steric stress between the phenyl ring that is attached to the N atom and the phenyl rings attached to the P atom. This stress causes the P atom to be pushed out of the plane and, therefore, **3B** isomer N-*trans*-CO has a higher energy than **3B** isomer O-*trans*-CO and is less stable. Using the electronic energies of the optimized molecules in the Boltzmann equation, 96.5% of **3A** isomer O-*trans*-CO and 100% of **3B** isomer O-*trans*-CO is predicted, in agreement with experimental observation. The rhodium(I) [Rh(N,O-BID)(CO)(PPh_3_)] complexes **3A** and **3B** are d^8^ complexes with electron occupation $d_{xy}{}^{2}d_{xz}{}^{2}d_{yz}{}^{2}d_{z^{2}}{}^{2}d_{x^{2}-y^{2}}{}^{0},$ see the selected d-based molecular orbitals (MOs) for [Rh(CH_3_COCHCNHCH_3_)(CO)(PPh_3_)] **3A** in Figure 2. The highest occupied molecular orbitals (HOMOs) of both **3A** and **3B** are mainly of rhodium $d_{z^{2}}$ character, see Figure 2. The $d_{z^{2}}$ HOMO plays an important role during the oxidative addition reaction between rhodium(I) complexes with CH_3_I [25,26], as will be discussed in Section 2.3.4 (below). 

#### 2.2.2. Rhodium(III) 

Oxidative addition to the two possible isomers of [Rh(CH_3_COCHCNHCH_3_)(CO)(PPh_3_)] **3A**, can theoretically lead to 12 possible Rh(III)-alkyl products (indicated with 1a–12a in Table 1, as well as their enantiomers 1b–12b) and 6 possible Rh(III)-acyl products (indicated with 13a–18a, as well as their enantiomers 13b–18b, in Table 1). The relative electronic energies of the optimized geometries of all the possible Rh(III) isomers that could be optimized are also given in Table 1. Some of the Rh(III)-acyl geometries were converted during the optimization to the geometries of the lowest energy Rh(III)-acyl isomers. The structures and DFT-calculated energies of the possible alkyl and acyl reaction products of the [Rh(CH_3_COCHCNPhCH_3_)(CO)(PPh_3_)] **3B** + CH_3_I reaction are given in Table 2. Since enantiomers have the same chemical properties and energies, we will only discuss the 12 possible Rh(III)-alkyl products (1a–12a) and 6 possible Rh(III)-acyl products (13a–18a). The energies obtained for the different Rh(III) products 1a–18a in Table 1 and Table 2 lead to the following insights:

- Products of [Rh(CH_3_COCHCNHCH_3_)(CO)(PPh_3_)] **3A** + CH_3_I reaction (Table 1): Evaluating the energies of the different Rh(III)-alkyl isomers in Table 1, it can be seen that the most preferred orientation of the product geometry is with the O_β-ketoiminato_ atom O atom of the ligand *trans* to CO (O-*trans*-CO) and the CH_3_ and I atoms bonded at the apical positions (top and bottom, with respect to the square planar geometry), since it has the lowest relative energy (2a, reaction product of CH_3_I and **3A** isomer O-*trans*-CO). The Rh(III)-alkyl isomer with the N_β-ketoiminato_ atom of the ligand *trans* to CO, and the CH_3_ and I atoms bonded at the apical positions (1a, reaction product of CH_3_I and **3A** isomer N-*trans*-CO) is only slightly higher in energy (0.07 eV). The energetically-preferred Rh(III)-alkyl isomer to form is, thus, 2a, while 1a may form in very small amounts. Evaluation of the energies of the Rh(III)-acyl isomers in Table 1, indicates that the two Rh(III)-acyl isomers 13a and 14a with the COCH_3_ ligand in the apical position have the lowest energy. Furthermore, the energy of Rh(III)-acyl isomers 13a and 14a are ca. 0.3 eV lower than the energy of the lowest energy Rh(III)-alkyl isomers 1a and 2a. This indicates that the Rh(III)-alkyl product of oxidative addition could convert, via CO insertion, to the lower more stable Rh(III)-acyl product as the final reaction product. Rh(III)-acyl 13a has slightly higher energy (0.04 eV) than 14a, therefore, the amount of 13a will be less than 14a.

**Table 1:** Calculated energies (using different DFT methods) and structures of the possible alkyl (1–12) and acyl (13–18) products of the oxidative addition reaction [Rh(CH_3_COCHCNHCH_3_)(CO)(PPh_3_)] **3A** + CH_3_I. In each cell the top entry gives PW91/TZP (PW91/TZP ZPE corrected in brackets), the middle entry TPSS-D3/TZ2P and bottom entry PBE-D3 DFT/TZ2P calculated energy.

Product	Structures	Relative Energy (eV)	Product	Structures	Relative Energy (eV)	Product	Structures	Relative Energy (eV)
**1a**	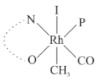	0.34 (0.29) 0.32 0.49	**7a**	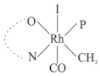	0.41 (0.35) 0.38 0.52	**13a**	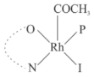	0.04 (0.04) 0.00 0.01
**1b**	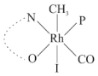	0.34 (0.29)	**7b**	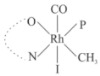	0.41 (0.35)	**13b**	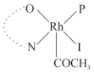	0.04 (0.04)
**2a**	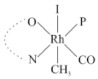	0.26 (0.21) 0.22 0.38	**8a**	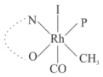	0.64 (0.58) 0.55 0.71	**14a**	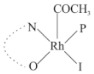	0.00 (0.03) 0.01 0.00
**2b**	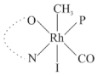	0.26 (0.19)	**8b**	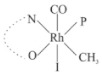	0.64 (0.58)	**14b**	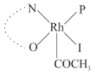	0.00 (0.00)
**3a**	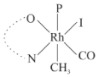	0.43 (0.34) 0.32 0.46	**9a**	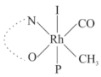	0.44 (0.37) 0.42 0.56	**15a**	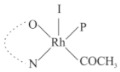	-
**3b**	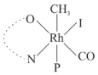	0.43 (0.35)	**9b**	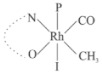	0.44 (0.37)	**15b**	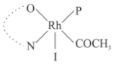	-
**4a**	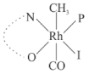	0.72 (0.64) 0.59 0.77	**10a**	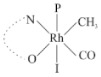	0.35 (0.28) 0.30 0.44	**16a**	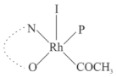	0.79 (0.72) 0.68 0.68
**4b**	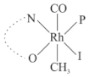	0.72 (0.63)	**10b**	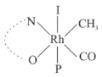	0.35 (0.28)	**16b**	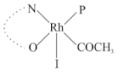	0.79 (0.70)
**5a**	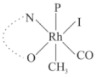	0.46 (0.37) 0.22 0.38	**11a**	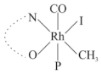	0.87 (0.74) 0.51 0.69	**17a**	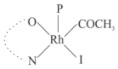	-
**5b**	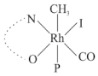	0.46 (0.37)	**11b**	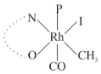	0.87 (0.74)	**17b**	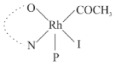	-
**6a**	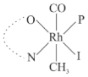	0.66 (0.57) 0.52 0.70	**12a**	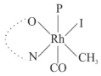	0.68 (0.59) 0.48 0.63	**18a**	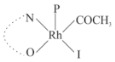	-
**6b**	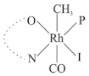	0.66 (0.57)	**12b**	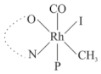	0.68 (0.62)	**18b**	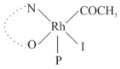	-

**Table 2:** Calculated energies (using different DFT methods) and structures of the possible alkyl (1–12) and acyl (13–18) products of the oxidative addition reaction [Rh(CH_3_COCHCNPhCH_3_)(CO)(PPh_3_)] **3B** + CH_3_I. In each cell, the top entry gives PW91/TZP (PW91/TZP ZPE, corrected in brackets), middle entry TPSS-D3/TZ2P, and bottom entry PBE-D3 DFT/TZ2P calculated energy.

Product	Structures	Relative Energy (eV)	Product	Structures	Relative Energy (eV)	Product	Structures	Relative Energy (eV)
**1a**	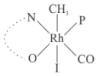	0.49 (0.50) 0.33 0.48	**7a**	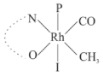	0.15 (0.17) 0.07 0.19	**13a**	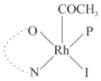	0.00 (0.08) 0.00 0.00
**1b**	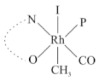	0.49 (0.51)	**7b**	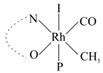	0.15 (0.16)	**13b**	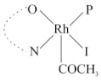	0.00 (0.08)
**2a**	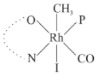	0.02 (0.00) 0.00 0.15	**8a**	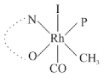	0.64 (0.64) 0.47 0.60	**14a**	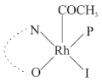	0.28 (0.33) 0.20 0.19
**2b**	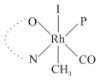	0.02 (0.03)	**8b**	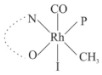	0.64 (0.64)	**14b**	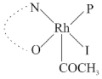	0.28 (0.31)
**3a**	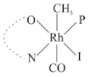	0.57 (0.58) 0.41 0.59	**9a**	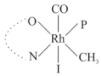	0.34 (0.32) 0.27 0.41	**15a**	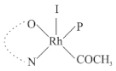	0.37 (0.35) 0.25 0.24
**3b**	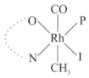	0.57 (0.56)	**9b**	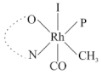	0.34 (0.34)	**15b**	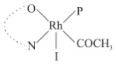	0.37 (0.35)
**4a**	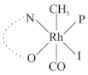	0.85 (0.85) 0.66 0.84	**10a**	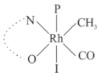	0.26 (0.27) 0.18 0.30	**16a**	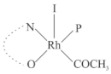	0.71 (-) 0.52 0.49
**4b**	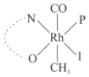	0.85 (0.84)	**10b**	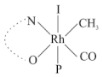	0.26 (0.27)	**16b**	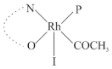	0.71 (-)
**5a**	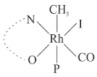	0.44 (0.43) 0.22 0.38	**11a**	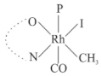	0.48 (0.51) 0.31 0.46	**17a**	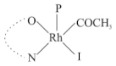	-
**5b**	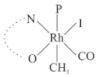	0.44 (0.41)	**11b**	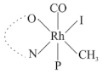	0.48 (0.51)	**17b**	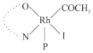	-
**6a**	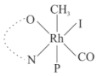	0.15 (0.15) 0.03 0.16	**12a**	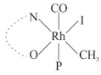	0.66 (0.61) 0.43 0.59	**18a**	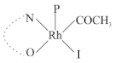	0.36 (0.40) 0.32 0.29
**6b**	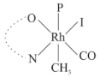	0.15 (0.41)	**12b**	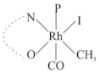	0.64 (0.66)	**18b**	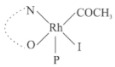	0.36 (0.38)

- Products of [Rh(CH_3_COCHCNPhCH_3_)(CO)(PPh_3_)] **3B** + CH_3_I reaction (Table 2): From the energies presented in Table 2, it is clear that Rh(III)-alkyl 2a and Rh(III)-acyl 13a are equi-energetic—in other words, there is no driving force for the Rh(III)-alkyl 2a to convert via CO insertion to the Rh(III)-acyl 13a. The Rh(III)-alkyl 2a product, with the O atom of the ligand *trans* to the CO, and the CH_3_ and I atoms, bonded at the apical positions (top and bottom with respect to the square planar geometry), are predicted to be the final reaction product of the [Rh(CH_3_COCHCNPhCH_3_)(CO)(PPh_3_)] **3B** + CH_3_I reaction.
- The [Rh(N,O-BID)(CO)(PPh_3_)] + CH_3_I oxidative addition reaction is theoretically only possible if the activation barrier of the transition state is low enough to form the product—in this case, a Rh(III)-alkyl product. Similarly the CO insertion reaction is theoretically only possible if there is a driving force to lower energy (to form the product) and the activation barrier of the transition state is low enough to form the Rh(III)-acyl product. A DFT study of the transition states will be presented in Section 2.3.4.

### 2.3. Kinetics of Iodomethane Oxidative Addition to Rhodium(I)

The oxidative addition reactions between the two [Rh(N,O-BID)(CO)(PPh_3_)] complexes **3A** and **3B** and CH_3_I are presented. The reaction is followed by IR, UV–Vis, and NMR. Additionally, a DFT study of the reaction will be presented to compliment the experimental findings. In each subsection, the results of **3B** will be presented first, followed by the results for **3A**, since it was experimentally found that **3B** exhibited a single reaction step, while the **3A** + CH_3_I reaction showed two steps.

#### 2.3.1. IR Study

The oxidative addition reaction was first followed on IR, since this technique is ideal to distinguish between CO bonds in metal-CO complexes of Rh(I)-carbonyl and Rh(III)-complexes. CO groups in Rh(I)-carbonyl complexes resonate at ~1980–2000 cm^−1^, CO groups in Rh(III)-alkyl-carbonyl complexes resonate at ~2050–2100 cm^−1^ and CO groups in Rh(III)-acyl complexes resonate at ~1700–1750 cm^−^^1^ [27].

**Scheme 2:**
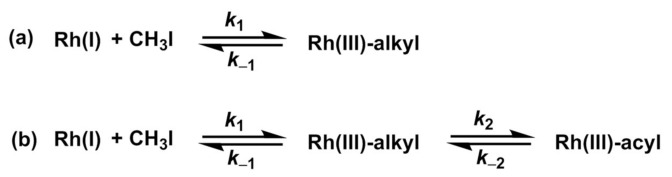
Reaction scheme for the (**a**) [Rh(CH_3_COCHCNPhCH_3_)(CO)(PPh_3_)] **3B** + CH_3_I reaction and (**b**) [Rh(CH_3_COCHCNHCH_3_)(CO)(PPh_3_)] **3A** + CH_3_I reaction.

**Figure 3:**
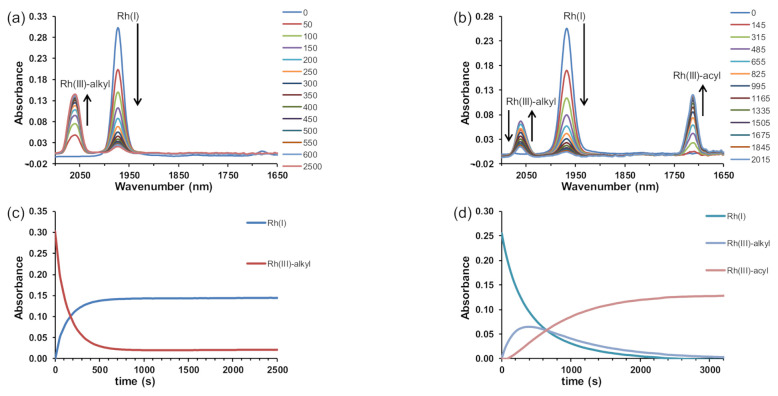
IR spectra of the reaction between (**a**) [Rh(CH_3_COCHCNPhCH_3_)(CO)(PPh_3_)] **3B** and 0.0272 mol dm^−3^ CH_3_I and (**b**) [Rh(CH_3_COCHCNHCH_3_)(CO)(PPh_3_)] **3A** and 0.0537 mol dm^−3^ CH_3_I, both at at 25 °C, with the CH_3_I concentration at 10× excess, with respect rhodium concentration at the indicated time in s. (**c**) The [Rh(CH_3_COCHCNPhCH_3_)(CO)(PPh_3_)] **3B** + CH_3_I reaction, as monitored at 1973.62 and 2059.72 cm^−1^, with time. (**d**) The [Rh(CH_3_COCHCNHCH_3_)(CO)(PPh_3_)] **3A** + CH_3_I reaction, as monitored at 1967.66, 2061.30 and 1713.74 cm^−1^, with time.

In Figure 3a, which displays the IR spectra of the CH_3_I reaction with [Rh(CH_3_COCHCNPhCH_3_)(CO)(PPh_3_)] **3B**, it can be seen that there are two main peaks that vary during the reaction. The first peak, at 1973.62 cm^−1^, is assigned to the disappearance of the Rh(I) complex as the reaction progresses and a product is formed, see Figure 3c. The second peak at 2059.72 cm^−1^ is assigned to the formation of the Rh(III)-alkyl reaction product. The rate constants determined from the IR kinetic experiments for different CH_3_I concentrations are given in Table 3. From Table 3, it can be seen that the rate of disappearance of [Rh(CH_3_COCHCNPhCH_3_)(CO)(PPh_3_)] **3B** and rate of the formation of the Rh(III)-alkyl product show very good correlation, since the values are so close to each other. This trend is seen across all three concentrations. The increase in the observed kinetic rate constant with the increase in CH_3_I concentration indicates that this reaction step is concentration-dependent. From the results obtained from the IR study, the reaction step of the [Rh(CH_3_COCHCNPhCH_3_)(CO)(PPh_3_)] **3B** + CH_3_I reaction can, thus, be presented as Scheme 2a.

In Figure 3b, which displays the IR spectra of the CH_3_I reaction with [Rh(CH_3_COCHCNHCH_3_)(CO)(PPh_3_)] **3A**, it can be seen that there are three main peaks that vary during the reaction. The Rh(I) complex displays a CO stretching frequency peak at 1967.66 cm^−1^. This absorbance peak decreases as the reaction progresses, which is explained by the disappearance of the Rh(I) complex, as it reacts with CH_3_I, in order to form a Rh(III) complex. The first product that forms is seen at the increase of the absorbance peak position at 2061.30 cm^−1^. This is attributed to the formation of a Rh(III)-alkyl complex after the direct addition of the CH_3_I to the Rh centre. This peak shows an increase at first, which shows the formation of the Rh(III)-alkyl product, up to a maximum absorbance level, after which it starts to decrease in intensity, see Figure 3d. This is attributed to the formation of a second Rh(III) product. When the Rh(III)-alkyl product starts to decrease in intensity, the formation of a second Rh(III) product can be seen at an absorbance peak of 1713.74 cm^−1^, see Figure 3b. The peak at 1713.74 cm^−1^ is designated to the Rh(III)-acyl complex. The intensity of the peak at 1713.74 cm^−1^ increases, up to a maximum amount, Figure 3d. The formation of the Rh(III)-acyl is due to CO insertion of the Rh(III)-alkyl product, and this step is called the second reaction step. Rh(III)-acyl is the final product observed for the [Rh(CH_3_COCHCNHCH_3_)(CO)(PPh_3_)] **3A** + CH_3_I reaction. The observed pseudo first-order reaction constant calculated at the indicated wavelengths for each reaction step is given in Table 3. From the data presented in Table 3 it can be seen that the observed rate constants for the loss of the [Rh(CH_3_COCHCNHCH_3_)(CO)(PPh_3_)] **3A** complex and the formation of the Rh(III)-alkyl product (the first reaction step), indicates a CH_3_I concentration-dependent step. The small differences between the rate constants for the Rh(I) loss and the Rh(III)-alkyl gain are due to the fact that the Rh(III)-alkyl is already converted to Rh(III)-acyl, while the first reaction step proceeds. Thus, the Rh(III)-alkyl increase during the first reaction step is, thus, virtually smaller [18]. The observed rate constants for the loss of the Rh(III)-alkyl product and formation of the Rh(III)-acyl product show good correlation with each other. There is barely any significant change in the rate values over the concentration range for the second step, which indicates that it is independent of the CH_3_I concentration. This is expected, since the conversion between the two products is not dependent on the concentration of CH_3_I, but on the individual properties of the complexes themselves, i.e., the type of ligand system employed. From the results obtained from the IR study, the reaction steps of the [Rh(CH_3_COCHCNHCH_3_)(CO)(PPh_3_)] **3A** + CH_3_I reaction can, thus, be presented as given in Scheme 2b.

#### 2.3.2. UV–Vis Study

The in situ analysis of the oxidative addition reaction between the [Rh(N,O-BID)(CO)(PPh_3_)], with N,O-BID = (CH_3_COCHCNHCH_3_)^−^ **1A** and (CH_3_COCHCNPhCH_3_)^−^ **1B**, complexes and CH_3_I are also measured by UV–Vis spectroscopy. Only the first step of both reactions, the [CH_3_I] dependent step, was followed on UV–Vis.

**Figure 4:**
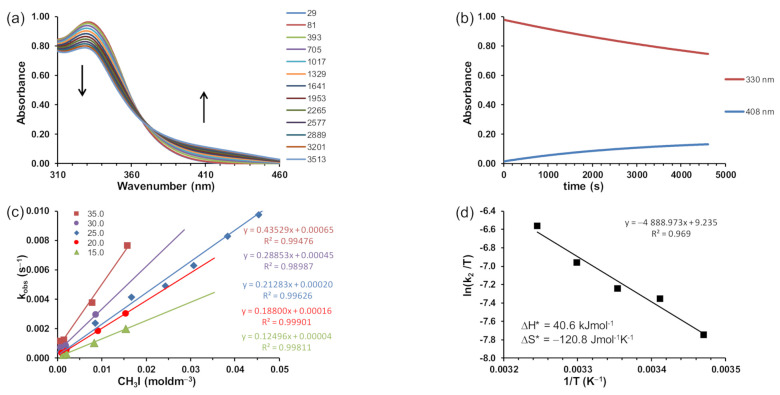
(**a**) UV–Vis spectra of the reaction between [Rh(CH_3_COCHCNHCH_3_)(CO)(PPh_3_)] **3A** and 0.008384 mol dm^−3^ CH_3_I concentration in 50× excess, with respect rhodium concentration at the indicated time in s. (**b**) The [Rh(CH_3_COCHCNHCH_3_)(CO)(PPh_3_)] **3A** + CH_3_I reaction, as monitored at the indicated wavelengths with time. (**c**) Graph of the observed kinetic rate constants against the concentrations of the reaction between [Rh(CH_3_COCHCNPhCH_3_)(CO)(PPh_3_)] **3B** and CH_3_I at 15, 20, 25, 30, and 35 °C, obtained from UV–Vis data at 404 nm. (**d**) Eyring plot of the **3B** and CH_3_I reaction in (**c**).

The reaction between the [Rh(CH_3_COCHCNHCH_3_)(CO(PPh_3_)] **3A** complex and CH_3_I yields spectra, as seen in Figure 4a. From Figure 4a, it is seen that there is a decrease of the absorption around a wavelength of 330 nm and increase of the absorption around 408 nm, as the reaction progresses. The decrease in the absorption (330 nm) is theorized to be the decrease in the concentration of the Rh(I) complex as it reacts with CH_3_I to form a Rh(III) complex. The increase in the absorption (408 nm) is identified as the Rh(III) product complex; however, the identification of the type of product is not possible. The Rh(III) products that are formed could either be the Rh(III)-alkyl, Rh(III)-acyl, or a mixture of both [17,28]. The absorbance changes, at both 330 nm and 408 nm, with time, are shown in Figure 4b.

The observed second-order reaction rate constants at different temperatures vs. the concentration of the CH_3_I in the oxidative addition reaction with [Rh(CH_3_COCHCNPhCH_3_)(CO)(PPh_3_)] **3B** are shown in Figure 4c. The slope of the different lines give the second-order rate constant for the oxidative addition reaction, with [Rh(CH_3_COCHCNPhCH_3_)(CO)(PPh_3_)] **3B** + CH_3_I at the indicated temperatures, summarized in Table 4, for the oxidative addition reaction of **3A** and **3B**. The activation enthalpy and entropy for the **3B** and CH_3_I reaction in Figure 4c can be obtained from the Eyring plot in Figure 4d. The large and negative activation entropy for the first oxidative addition step is indicative of an associative mechanism, as commonly found for oxidative addition of CH_3_I to square planar d^8^ metal complexes, which is proposed to proceed via an S_N_2 mechanism [26,29,30,31].

#### 2.3.3. NMR Study

The UV–Vis spectrophotometric analysis yielded the second-order rate constants for the two phosphine-containing Rh complexes, and the IR spectrophotometric analysis yielded information regarding the types of products formed (alkyl and/or acyl) during the reaction between the Rh(I) complexes and CH_3_I. In order to gather more information, regarding the types of products and possible isomers involved in the reaction, an in situ analysis of the reaction was performed through the use of ^1^H-NMR spectroscopy. All possible product structures, of which there are multiples for each product type, will be presented and discussed in this section.

**Figure 5:**
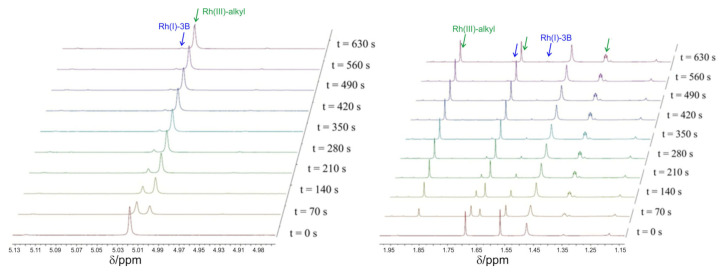
Stacked ^1^H-NMR spectra of the reaction between the [Rh(CH_3_COCHCNPhCH_3_)(CO)(PPh_3_)] **3B** complex and CH_3_I, focusing on the methine (**left**) and methyl (**right**) proton signals at 25 °C. The CH_3_I concentration (0.0502 mol dm^−3^) was in a 10× excess.

The [Rh(CH_3_COCHCNPhCH_3_)(CO)(PPh_3_)] **3B** and CH_3_I reaction yielded similar results, as with the IR study, in that there was only one product formed during the reaction, namely the Rh(III)-alkyl product. Only one Rh(I) reactant **3B** and one Rh(III)-alkyl product isomer was observed on NMR. The methine and methyl proton signals were tabulated in Table 5, with the ^1^H-NMR region presented in Figure 5. From Figure 5 (left), it can be seen that the resonance peak for the methine proton of the starting [Rh(CH_3_COCHCNPhCH_3_)(CO)(PPh_3_)] **3B** complex (5.018 ppm) decreased as the reaction progressed, and a resonance peak, representing the methine proton signal for the Rh(III)-alkyl product (5.004 ppm), increased during the course of the reaction [17,18,28]. Figure 5 (right) illustrates the change in the resonance peak intensities as the reaction progressed, with respect to the methyl proton signals. The [Rh(CH_3_COCHCNPhCH_3_)(CO)(PPh_3_)] **3B** methyl resonances (blue arrows, 1.688 ppm and 1.567 ppm) decreased as the reaction proceeded, and the Rh(III)-alkyl product that formed has methyl resonances (green arrows, 1.869 ppm and 1.655 ppm) that increased as the reaction proceeded. The doublet of doublets that formed (~1.370 ppm) is attributed to the CH_3_ of the CH_3_I, attached to the Rh central atom (formation of an alkyl reaction product). The doublet of doublets signal of the CH_3_ group of the alkyl was due to coupling with Rh (spin ½) and P (spin ½) [18,28]. This doublet of doublets signal also increased as the reaction proceeded. Figure 5 show that only one isomer of the Rh(III)-alkyl product formed during the reaction from a single isomer of **3B**. Table 5 contains the observed pseudo first-order reaction rate constants, obtained from the change in concentration with time, of both the methine and methyl resonance signals. The observed kinetic rate constants, obtained from the methine and methyl peaks, when compared with those obtained through UV–Vis and IR spectroscopy, show good correlation between the values, see Table 3, Table 4 and Table 5.

**Figure 6:**
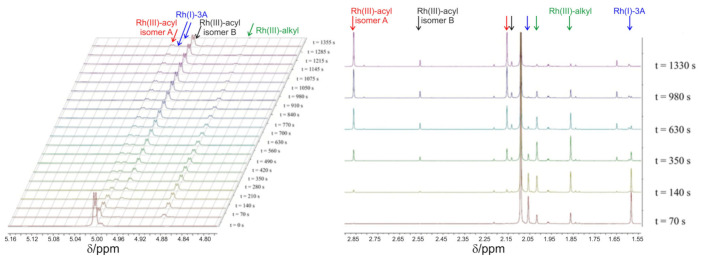
Stacked ^1^H-NMR spectra of the reaction between the [Rh(CH_3_COCHCNHCH_3_)(CO)(PPh_3_)] **3A** complex and CH_3_I, focusing on the methine (**left**) and methyl (**right**) proton signals at 25 °C. The CH_3_I concentration (0.0301 mol dm^−3^) was in a 6× excess.

**Figure 7:**
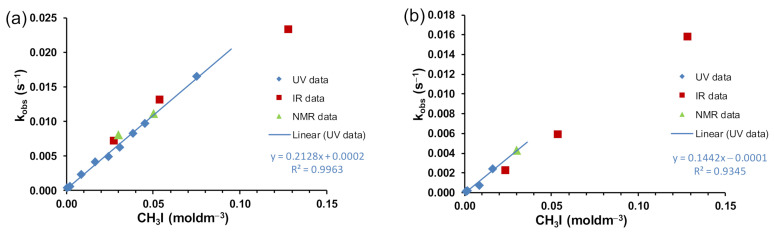
Observed kinetic rate constants for obtained through UV–Vis spectroscopy, IR spectroscopy, and NMR spectroscopy, at 25 °C, for the reaction between CH_3_I and (**a**) [Rh(CH_3_COCHCNHCH_3_)(CO)(PPh_3_)] **3B** and (**b**) [Rh(CH_3_COCHCNPhCH_3_)(CO)(PPh_3_)] **3A**.

The [Rh(CH_3_COCHCNHCH_3_)(CO)(PPh_3_)] **3A** and CH_3_I reaction yielded similar behaviour to those observed in the IR study, in that there were two reaction steps observed during the reaction on NMR, according to Scheme 2b. The methine and methyl proton signals are tabulated in Table 5, with the ^1^H-NMR region presented in Figure 6. Figure 6 (left) illustrates the methine proton signals as the reaction progressed. The pure [Rh(CH_3_COCHCNHCH_3_)(CO)(PPh_3_)] **3A** isomer A (5.002 ppm) and isomer B (4.990 ppm) showed a decrease in the proton signal intensity as the reaction progressed, with the formation of Rh(III)-alkyl appearing at 4.881 ppm. Only one Rh(III)-alkyl isomer was observed, but the existence of a second Rh(III) isomer cannot be excluded. The second Rh(I) isomer B is minute; therefore, the alkyl that could formed from the small Rh(I) isomer B could not be observed, since the peak was too small, or possibly due to overlap with the Rh(III)-alkyl isomer observed. The resonance peaks of the Rh(III)-alkyl isomers increased for a short time during the reaction, up to a maximum intensity, after which it starts to decrease. This is due to the formation of the Rh(III)-acyl product isomers (4.976 ppm and 5.014 ppm), which caused a decrease in the Rh(III)-alkyl isomers’ signal intensity. It was observed that the intensity of the signals, due to the second Rh(III)-acyl isomer B, was very small and, although observed, the formation of the second Rh(III)-acyl isomer B could not be followed kinetically [26,27,32]. Figure 6 (right) displays the change in the methyl proton signals between the two isomers of the starting material, [Rh(CH_3_COCHCNHCH_3_)(CO)(PPh_3_)] **3A**, and alkyl product that form as the reaction progresses. The Rh(I) starting material isomers (blue arrows, 2.056 ppm and 1.583 ppm, main isomer) showed a decrease in the two methyl proton signals, and Rh(III)-alkyl product (green arrows, 2.017 ppm and 1.862 ppm) showed an increase in the signal strength at first. As the reaction progressed, signals of the Rh(III)-alkyl product isomers decreased, and the Rh(III)-acyl product isomers (main isomer A, pink arrows, 2.861 ppm and 2.157 ppm and minor isomer B, black arrows, 2.555 ppm and 2.133 ppm) showed an increase in the signal strength as time progresses. The reason for the Rh(III)-alkyl methyl proton resonances showing an increase, and then a decrease, is the same as for the methine proton focussed analysis that is: the Rh(III)-alkyl product starts forming up to a maximum, after which it starts to convert to the Rh(III)-acyl product [27,32]. Table 5 contains the observed pseudo first-order reaction rate constants of both the methine and methyl resonance signals. The observed kinetic rate constants, obtained from the methine and methyl peaks, when compared with those obtained through UV–Vis and IR spectrophotometry, showed a good correlation between the values, as seen in Figure 7b.

#### 2.3.4. DFT Study of Reaction Mechanism

**Figure 8:**
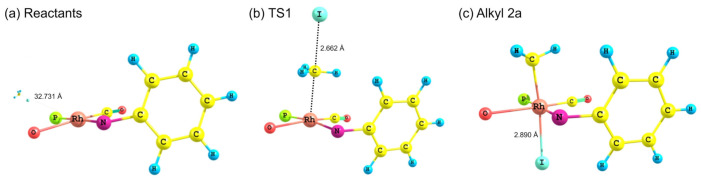
(**a**) DFT-calculated optimized geometries of the [Rh(CH_3_COCHCNPhCH_3_)(CO)(PPh_3_)] **3B** complex (isomer O-*trans*-CO) and CH_3_I, with a large separation between the reactant molecules, referred to as reactants. (**b**) DFT-calculated transition state of the linear oxidative addition reaction between the [Rh(CH_3_COCHCNPhCH_3_)(CO)(PPh_3_)] **3B** and CH_3_I. Referred to as TS1. The bonds, associated with the imaginary frequency of the transition state, are indicated with black dotted lines. (**c**) DFT-calculated optimized geometry of the Rh(III)-alkyl 2a product, referred to as Alkyl 2a. The three Ph groups of the PPh_3_ ligand and CH_3_CCHCCH_3_ backbone of [CH_3_COCHCNPhCH_3_] ligand are excluded for clarity.

A DFT study of the reactants, transition state, and the products of the [Rh(CH_3_COCHCNPhCH_3_)(CO)(PPh_3_)] **3B** + CH_3_I oxidative addition reaction is presented in Figure 8, with the results summarized in Table 6 (results for the most stable O-*trans*-CO isomer of **3B** is presented). Figure 8a contains the optimized geometries of the reactant molecule, [Rh(CH_3_COCHCNPhCH_3_)(CO)(PPh_3_)] **3B**, complex’s reaction with CH_3_I, before the reaction starts. There was a large separation between the reactant molecules (d = 32.731 Å) at the moment of the mixture. The reactant molecules then move through the bulk solution, until the distance between them is short enough and orientation of the molecules is sufficient to facilitate a reaction to occur [26,32]. The oxidative addition reaction between CH_3_I and square planar Rh(I) complexes can theoretically proceed via three different transition state structures [33,34,35]. The three methods of addition are a linear/back addition (where there is a near 180° angle formed between the approaching CH_3_I and Rh (*trans* addition)), bent addition (where the angle is between 90° and 180° (*trans* addition)), and front addition (where the angle is less than 90° (*cis* addition)). DFT computations showed that the most preferred addition method was a linear/back approach of the CH_3_I to the Rh(I) metal centre. This method had an activation barrier that was ~7 times lower in energy than the other two methods [26]. Figure 8b contains the optimized geometry of the linear oxidative addition of the methyl group to the Rh(I) metal centre of the [Rh(CH_3_COCHCNPhCH_3_)(CO)(PPh_3_)] **3B** complex. The CH_3_I molecule, when it is close enough to the Rh complex molecule, approaches linearly from the apical position (top as illustrated), with the methyl group closest to the Rh complex. This approach of the CH_3_I molecule, and subsequent bond formation between the methyl group and Rh metal centre, cause an increase in the total system energy, up to a maximum value, the activation energy. After the Rh-C_CH3I_ bond is formed, the energy of the system decreases again, as the bond formed stabilizes and the I^−^ ion diffuses away [26,32]. The I^−^ ion is then free to diffuse away into the bulk solution to react with another five-coordinate Rh complex. The bond formation between the five-coordinate cationic intermediate and a I^−^ ion is a barrierless process, with the formation of the Rh(III)-alkyl 2a product with the O_β-ketoiminat_ atom of the ligand *trans* to CO, and the CH_3_ and I atoms bonded at the apical positions (top and bottom with respect to the square planar geometry), see Figure 8c. The energies of the reaction steps calculated by DFT are summarized in Table 6. The energies are given relative to the final product in the reaction mechanism. The energy barrier of the transition state (TS) is 0.29 eV and 0.83 eV higher than the reactants and [Rh(CH_3_COCHCNPhCH_3_)(CO)(PPh_3_)(CH_3_)(I)]-alkyl 2a product, respectively. The energy profile is, thus, favourable for the product formation. All DFT attempts at determining a TS for formation of an acyl product through CO insertion at the Rh(III)-alkyl 2a failed. This fact, as well as the fact that Rh(III)-alkyl 2a and Rh(III)-acyl 13a are equi-energetic (see energies in Table 2), with no driving force for CO insertion to occur in 2a to form 13a, compliment the experimental findings that a Rh(III)-alkyl is the final reaction product of the [Rh(CH_3_COCHCNPhCH_3_)(CO)(PPh_3_)] **3B** + CH_3_I reaction.

**Figure 9:**
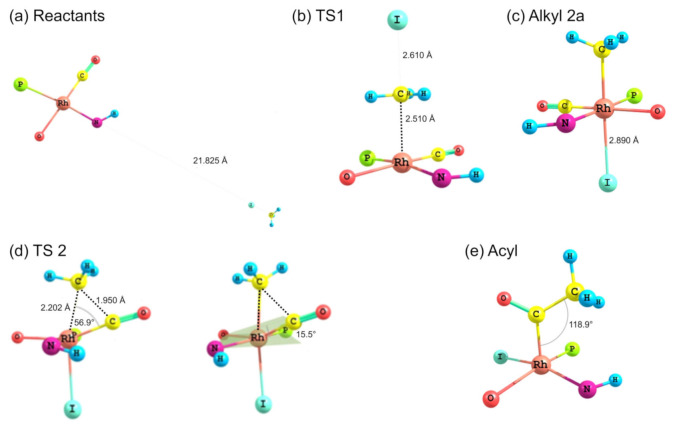
(**a**) DFT-calculated optimized geometries of the [Rh(CH_3_COCHCNHCH_3_)(CO)(PPh_3_)] **3A** (isomer O-*trans*-CO) complex and CH_3_I with a large separation between the reactant molecules, referred to as Reactants. (**b**) DFT-calculated optimized geometry of the linear oxidative addition reaction between the [Rh(CH_3_COCHCNHCH_3_)(CO)(PPh_3_)] **3A** and CH_3_I. The methyl group of the CH_3_I moves towards the Rh metal centre in order to form a bond and the distance between the methyl group and the I atom increases, referred to as TS1. (**c**) DFT-calculated optimized geometry of the Rh(III)-alkyl 2a product formed during step 1, referred to as Alkyl-2a. (**d**) Left: Geometry of the DFT-calculated transition state of the methyl migration towards the CO group in the formation of the [Rh(CH_3_COCHCNHCH_3_)(CO)(PPh_3_)] **3A** acyl 14a product. Right: Deviation of the CO group from the square planar geometry, referred to as TS2. (**e**) DFT-calculated optimized geometry of the [Rh(CH_3_COCHCNHCH_3_)(CO)(PPh_3_)]-acyl product, after the migration of the methyl group towards the CO group, referred to as Acyl. The three Ph groups of the PPh_3_ ligand and CH_3_CCHCCH_3_ backbone of [CH_3_COCHCNHCH_3_] ligand were excluded for clarity. The bonds associated with the imaginary frequency of TS1 and TS2 are indicated with black dotted lines.

The DFT-calculated results for the [Rh(CH_3_COCHCNHCH_3_)(CO)(PPh_3_)] **3A** + CH_3_I oxidative addition reaction are presented in Figure 9, with the results summarized in Table 6. The results related to the most stable reactant isomer, the isomer with O_β-ketoiminato_ *trans* to CO (O-*trans*-CO), is presented. Figure 9a contains the optimized geometries of the reactant molecules, [Rh(CH_3_COCHCNHCH_3_)(CO)(PPh_3_)] **3A** and CH_3_I, before the oxidative addition reaction proceeds. There is a large separation between the two molecules. The molecules merely move through the bulk solution until both the distance between the two is short enough and the orientation of the molecules is correct in order to facilitate a reaction. There is a distance between the two molecules >21.825 Å. As the molecules move through the bulk solution this distance decreases.

Figure 9b contains the optimized geometries of the first TS of the [Rh(CH_3_COCHCNHCH_3_)(CO)(PPh_3_)] **3A** + CH_3_I reaction. The CH_3_I approaches the [Rh(CH_3_COCHCNHCH_3_)(CO)(PPh_3_)] **3A** complex from the apical position linearly with the methyl group closest to the Rh metal centre. As the CH_3_I molecule approaches, the distance between the methyl group and I increases from 2.1736 Å (DFT optimized geometry of CH_3_I) to 2.610 Å, and the distance between the methyl group and Rh metal centre decreases, until a bond is formed. When the distance between the methyl group and the I atom reaches a certain length the bond between the two breaks, and the I^−^ ion is then free to diffuse back into the bulk solution in order to react with another five-coordinate Rh intermediate that is formed. As the Rh-C_CH3I_ distance decreases, the total energy of the system increases up to a maximum point. At this maximum the transition state occurs, where the CH_3_I molecule breaks up into a methyl group and I^−^ ion, and the methyl group then forms a bond with the Rh metal centre to form a five-coordinate intermediate complex as seen in Figure 9b. After the bond is formed, the total system energy then starts to decrease. As the I^−^ ion approaches the Rh metal centre, the total energy of the system decreases, up until the bond formation occurs, after which the system reaches a point of stability, as shown in Figure 9c, with the formation of the Rh(III)-alkyl complex.

The next reaction step is the CO insertion of the CH_3_ migration step. The CH_3_ group, thus, starts moving towards the C atom of the CO group. As the CH_3_ migrates towards the CO group, the CO group gets lifted out of the plane formed between the N and O atoms of the ligand and P atom of the PPh_3_ ligand by 15.50°—effectively decreasing the C_methyl_-Rh-C_co_ bond angle even more. During the migration the Rh-C_methyl_ bond length increases, and the C_methyl_-C_co_ distance decreases. These changes continue up to a certain point (C_methyl_-Rh-C_co_ = 56.92°), where the Rh-C_methyl_ bond breaks and the C_methyl_-C_co_ bond forms [26,32]. As can be seen from Figure 9d, when the moment of the transition state is reached, the ligand starts shifting or flipping, in order to position the COCH_3_ group in an apical position. Figure 9e illustrates the final product’s geometry, optimized by DFT calculations, of the [Rh(CH_3_COCHCNHCH_3_)(CO)(PPh_3_)]-acyl product 14a. It can be seen that the acyl group is now in an apical position and I atom has taken the place of the PPh_3_ group from the [Rh(CH_3_COCHCNHCH_3_)(CO)(PPh_3_)]-alkyl product 2a, with the PPh_3_ group taking the place of the CO group of alkyl product 2a. This is due to this orientation having a smaller total system energy than any of the other orientations. After the bond is formed between the C_methyl_ and the C_CO_ atoms, the orientation starts to change. The six-membered ring, formed between the Rh metal centre and the L,L′-BID ligand, flips. The O atom moves in such a way as to allow for the I atom to be positioned *trans* to the N atom. During this shift the energy of the system decreases up to a minimum where the I atom is *trans* and no longer in an apical position until the final, stable geometry of the five-coordinate Rh(III) complex was obtained [26,32]. 

The energies of the two reaction steps (calculated by DFT) are summarized in Table 6. The energies are given relative to the final product in the reaction mechanism. From Table 6 it can be seen that the first transition state, the linear addition of CH_3_I, to the structure has an energy barrier of only 0.26 eV. This energy barrier calculated for the [Rh(CH_3_COCHCNHCH_3_)(CO)(PPh_3_)] **3A** + CH_3_I reaction, is favourable for the reaction to occur and to proceed to the Rh(III)-alkyl reaction product that is 0.82 eV lower in energy than the TS1. The energy barrier of the second step is higher than the first step (0.91 eV vs. 0.26 eV), indicating that the second reaction is much slower than the first reaction step, in agreement with experimental results. The Rh(III)-acyl product has the lowest energy and, thus, most stable and constitutes the final reaction product. These computational results are, thus, corroborated by both the IR and NMR spectra illustrating the formation of an alkyl and acyl product, with the acyl product being the final product. 

#### 2.3.5. Summary of [Rh(L,L′-BID)(CO)(PPh_3_)] + CH_3_I Reactions

**Scheme 3:**
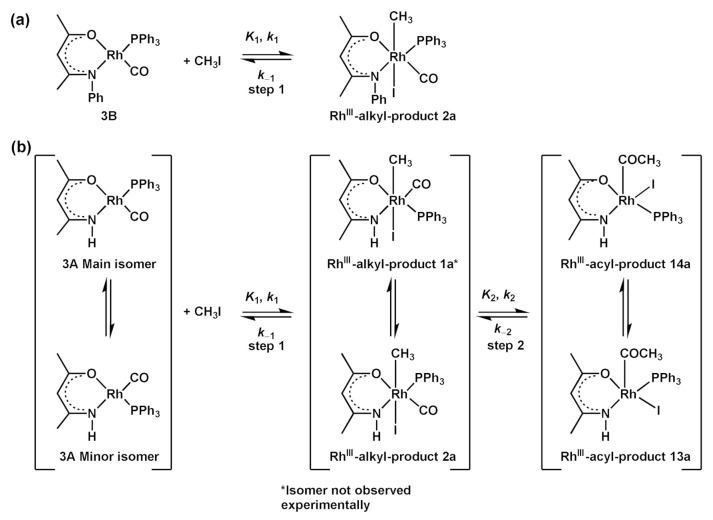
Reaction scheme for the oxidative addition reaction of (**a**) [Rh(CH_3_COCHCNPhCH_3_)(CO)(PPh_3_)] **3B** and CH_3_I and (**b**) [Rh(CH_3_COCHCNHCH_3_)(CO)(PPh_3_)] **3A** and CH_3_I.

In Figure 7 the results for the second-order reaction rate constants of the [Rh(CH_3_COCHCNPhCH_3_)(CO)(PPh_3_)] **3B** + CH_3_I reaction and the [Rh(CH_3_COCHCNHCH_3_)(CO)(PPh_3_)] **3A** + CH_3_I reaction, respectively (as obtained by IR, UV–Vis and NMR), are compared. The second-order reaction rate constants obtained are comparable within experimental error. These results, along with the DFT-calculated results, suggest a one-step reaction, leading from [Rh(CH_3_COCHCNPhCH_3_)(CO)(PPh_3_)] **3B** and CH_3_I, as reactants, to a [Rh(CH_3_COCHCNPhCH_3_)(CO)(PPh_3_)(CH_3_)(I)] alkyl product 2a, with no additional steps taking place in the reaction. The reaction scheme for this complex can be seen in Scheme 3a. The results obtained regarding the different reaction products from IR and NMR, along with the DFT-calculated results, suggest a two-step reaction leading from [Rh(CH_3_COCHCNHCH_3_)(CO)(PPh_3_)] **3A** (both isomers) and CH_3_I as reactants, to two [Rh(CH_3_COCHCNHCH_3_)(CO)(PPh_3_)(CH_3_)(I)] alkyl products (2a and 1a, 1a currently not observed experimentally), to the formation of two [Rh(CH_3_COCHCNHCH_3_)(PPh_3_)(COCH_3_)(I)] acyl products (14a and 13a). The reaction scheme for this complex can be seen in Scheme 3b.

Considering the [Rh(L,L′-BID)(CO)(PPh_3_)] + CH_3_I oxidative addition reaction, where L,L′-BID = O,O-BID, a β-diketonato ligand (RCOCHCOR′)^−^, it is found that for more electronegative the groups R and R′ attached to the backbone of the ligand, a slower oxidative addition reaction rate is obtained [11,36]. For example, the reaction rate for the oxidation addition step of the [Rh(CF_3_COCHCOCF_3_)(CO)(PPh_3_)] + CH_3_I reaction (0.00013(1) mol^−1^ dm^3^ s^−1^ [37]) is more than 100 times smaller the [Rh(CH_3_COCHCOCH_3_)(CO)(PPh_3_)] + CH_3_I reaction (0.024(3) mol^−1^ dm^3^ s^−1^ [38]). This is because the two CF_3_ groups attached to the ligand in [Rh(CF_3_COCHCOCF_3_)(CO)(PPh_3_)] are more electronegative than the two CH_3_ groups ($\chi_{CF_{3\;}}=3.01\;\mathrm{and}\;\chi_{CH_{3\;}}=3.34\;$ [25]) in [Rh(CH_3_COCHCOCH_3_)(CO)(PPh_3_)].

The N,O-BID containing [Rh(CH_3_COCHCNRCH_3_)(CO)(PPh_3_)] complexes **3A** (R = H) and **3B** (R = Ph) of this study can be compared with the O,O-BID-containing [Rh(CH_3_COCHCOCH_3_)(CO)(PPh_3_)] complex. In agreement with the larger electronegativity of O (3.44 Pauling scale) than that of N (3.04 on the Pauling scale), it was found that the oxidative addition reaction rate of CH_3_I to [Rh(CH_3_COCHCOCH_3_)(CO)(PPh_3_)] (0.024(3) mol^−1^ dm^3^ s^−1^ [38]) is slower than the oxidative addition reaction rate of CH_3_I to **3A** and **3B**, 0.144(1) mol^−1^ dm^3^ s^−1^ and 0.213(5) mol^−1^ dm^3^ s^−1^, respectively. The more electronegative O atom in [Rh(CH_3_COCHCOCH_3_)(CO)(PPh_3_)] withdraws more electron density from it’s $d_{z^{2}}$, HOMO, than the N atom in **3A** and **3B** (Figure 2). The nucleophilic attack of the rhodium(I) centre on the CH_3_ of the CH_3_I group, thus, proceeds slower in the case of [Rh(CH_3_COCHCOCH_3_)(CO)(PPh_3_)] than for **3A** and **3B**.

## 3. Materials and Methods

### 3.1. Instrumentation

Characterization of the complexes were performed on a Bruker Avance DPX 300 NMR (Bruker, Hanau, Germany) utilizing CDCl_3_ as solvent at 298 K. Kinetic measurements were performed on a Bruker Avance 600 MHz NMR (Bruker, Hanau, Germany), utilizing CDCl_3_, passed through basic alumina, as solvent and the CH_3_I at concentrations of 10 times in excess.

Characterization of the complexes were carried out with a Bruker Tensor 27 FTIR infrared spectrophotometer (Bruker, Hanau, Germany) fitted with a Pike MIRacle single-bounce and diamond ATR. Kinetic measurements of the [Rh(N,O-BID)(CO)(PPh_3_)] complexes were carried out on a Bruker Tensor 27 FTIR infrared spectrophotometer (Bruker, Hanau, Germany), fitted for solution state analysis with a Rh complex concentration 0.005 mol dm^−3^, and the CH_3_I concentration varied between 10, 20, and 50 times in excess. Chloroform, passed through basic alumina, was utilized as solvent, and all measurements were performed at 25 °C.

Kinetic measurements of the [Rh(N,O-BID)(CO)(PPh_3_)] complexes were carried out with a Shimadzu UV-1650PC UV–Vis spectrophotometer (Shimadzu, Kyoto, Japan), fitted with a CPS-240A cell positioner and temperature controller. Chloroform, passed through basic alumina, was utilized as solvent and the Rh complex concentrations were 0.0003 mol dm^−3^, with the concentration of the CH_3_I at 5, 10, 50, and 100 times in excess. Measurements were taken at the temperatures indicated.

The ADF program (Software for Chemistry & Materials, Amsterdam, The Netherlands) [39] was utilized in the calculations of the complexes. A TZP basis set with the PW91 [40] functional was utilized in the optimization of the geometries. The theoretical IR frequencies were calculated with the selected basis set and functional to obtain zero point energy (ZPE) corrected electronic energies. Solvent effects were taken into account using chloroform as solvent, using the COSMO [41,42,43] model of solvation with an Esurf cavity [44]. Electronic energies for the rhodium(III) products were also calculated, using the optimized PW91 geometries and the TPSS [45,46] and PBE [47] functionals, respectively, both including Grimme’s D3 [48] dispersion correction and the TZ2P basis set. The optimized coordinates of the DFT calculations are provided in Supplementary Materials.

### 3.2. Synthesis

The synthesis of the ligands **1A** and **1B** was performed as published in literature [15].

#### 3.2.1. [Rh(N,O-BID)(CO)_2_]

The synthesis of [Rh(CH_3_COCHCNHCH_3_)(CO)_2_] **2A** and [Rh(CH_3_COCHCNPhCH_3_)(CO)_2_] **2B** was performed as published in literature for **2B** [15] and related complexes [16]. Di-µ-chloro-tetracarbonyldirhodium(I) (0.05 g, 0.1286 mmol) was dissolved in methanol (5 mL), and the ligand (0.2572 mmol), also dissolved in methanol (2 mL), was added dropwise over 10 min whilst stirring. The mixture was left to stir for 1 h. The mixture was then extracted with *n*-hexane, until the *n*-hexane was clear, solvents combined, and reduced under reduced pressure. The solid that precipitated out was then collected, recrystallized, and weighed.

Characterization data:

[Rh(CH_3_COCHCNHCH_3_)(CO)_2_] **2A**: Yield = 69%. ^1^H-NMR: 5.3635–5.2933 ppm (d, C-H, *J*^4^ = 2.311 Hz); 2.1749–2.1720 ppm (d, CH_3_-CN, *J*^4^ = 0.74 Hz); 2.1060 ppm (CH_3_-CO). ν_CO_: 2044.13 cm^−1^; 1971.02 cm^−1^.

[Rh(CH_3_COCHCNPhCH_3_)(CO)_2_] **2B**: Yield = 78%. ^1^H-NMR: 7.3882–7.0605 ppm (m, C_6_H_5_-N); 5.2977 ppm (s, C-H); 2.1949 ppm (s, CH_3_-CN); 2.1414 ppm (s, CH_3_-CO). ν_CO_: 2058.98 cm^−1^; 1997.69 cm^−1^. 

#### 3.2.2. [Rh(N,O-BID)(CO)(PPh_3_)]

[Rh(N,O-BID)(CO)_2_] (0.2 mmol) was dissolved in *n*-hexane (3 mL). Triphenylphosphine (0.23 mmol) was dissolved in *n*-hexane (3 mL) and added dropwise over 10 min to the dicarbonyl complex solution whilst stirring. The mixture was then left to stir, until no more bubbles evolved. The precipitate that formed was filtered off, recrystallized, and weighed. 

Characterization data:

**3A**: Yield = 77%. ^1^H-NMR: Isomer A: 7.7153–7.3524 ppm (m, C_6_H_5_-P); 5.1005–5.0925 ppm (d, C-H, *J*^4^ = 0.93 Hz); 2.1520–2.1490 ppm (d, CH_3_-CN, *J*^4^ = 2.43 Hz); 1.6758 ppm (s, CH_3_-CO). Isomer B: 7.7153–7.3524 ppm (m, 3 × C_6_H_5_-P); 5.0635 ppm (s, C-H); 2.0617 ppm (s, CH_3_-CN); 1.9335 ppm (s, CH_3_-CO). ν_CO_: 1952.67 cm^−1^.

**3B**: Yield = 65%. ^1^H-NMR: 7.3890–7.0608 ppm (m, C_6_H_5_-N; 3 × C_6_H_5_-P); 5.2982 ppm (s, C-H); 2.1417 ppm (s, CH_3_-CN); 1.7852 ppm (s, CH_3_-CO). ν_CO_: 1966.60 cm^−1^.

### 3.3. Kinetics

Oxidative addition reactions between the triphenylphosphine-containing Rh complexes, **3A** and **3B**, and iodomethane, CH_3_I, were monitored on UV–Vis spectrophotometry, IR spectrophotometry and ^1^H-NMR spectrometry, as described in our previous publications [11,36]. All kinetic measurements were performed under the indicated pseudo first-order conditions (CH_3_I being in excess, with respect to the concentration of the rhodium complex). The observed first-order rate constants *k*_obs_ were obtained from the slope of the graph of ln(A_inf_ − A_t_) vs. time data, according to the first-order kinetic equation:

ln(A_inf_ − A_t_) = −*k*_obs_t + ln(A_inf_ − A_0_)

where A_t_ = absorbance of a complex at time t, A_inf_ and A_0_ is absorbance of complex at time infinite and zero, respectively. The second-order rate constants, *k*_2_ (in dm^3^ mol^−1^ s^−1^), were obtained from the slope of the graph of *k*_obs_ against the concentration of CH_3_I:

*k*_obs_ = *k*_2_[CH_3_I] + *k_−_*_2_

where *k*_2_ (*k_−_*_2_) is the second-order rate constant of the forward (backward) reaction of the kinetic reaction between the Rh complexes **3A** and **3B** and CH_3_I.

Activation parameters were obtained from the Eyring relationship:

$$ln\frac{k_{1}}{T}=-\frac{∆H^{\#}}{\mathrm{RT}}+\frac{∆S^{\#}}{R}+ln\frac{k_{B}}{h}$$

where ∆*H*^#^ = activation enthalpy, ∆*S*^#^ = activation entropy, T = temperature, *k*_2_ = second-order rate constant of the first kinetic step at temperature T, *k*_B_ = Boltzmann’s constant, *h* = Planck’s constant, R = universal gas constant.

## 4. Conclusions

Experimentally, one isomer of [Rh(CH_3_COCHCNPhCH_3_)(CO)(PPh_3_)] **3B** and two isomers of [Rh(CH_3_COCHCNHCH_3_)(CO)(PPh_3_)] **3A** were observed. DFT calculations showed that the **3B** isomer N-*trans*-CO exhibited a distorted square planar geometry, due to the steric stress between the phenyl ring, which is attached to the N atom and phenyl rings attached to the P atom, causing the **3B** isomer N-*trans*-CO to be less stable. The electronic energies of the optimized molecules, in the Boltzmann equation, predicted 100% of **3B** isomer O-*trans*-CO (no **3B** isomer N-*trans*-CO) and 96.5% of **3A** isomer O-*trans*-CO, in agreement with experimental observation.

Experimentally, the [Rh(CH_3_COCHCNPhCH_3_)(CO)(PPh_3_)] (**3B**) + CH_3_I reaction proceeds through one reaction step, with a rhodium(III)-alkyl as final reaction product. The DFT calculations of all the possible reaction products and transition states, in agreement with experimental findings, showed that, for the experimentally observed one-step reaction [Rh(CH_3_COCHCNPhCH_3_)(CO)(PPh_3_)] (**3B**) + CH_3_I, the lowest energy Rh(III)-alkyl 2a and Rh(III)-acyl 13a reaction products are equi-energetic. There is, thus, no driving force for CO insertion to occur in 2a to form 13a.

Experimentally, the [Rh(CH_3_COCHCNHCH_3_)(CO)(PPh_3_)] (**3A**) + CH_3_I reaction proceeds through two reaction steps, with rhodium(III)-acyl as the final reaction product. The DFT calculations of all the possible reaction products and transition states, in agreement with experimental findings, showed that, for the experimentally observed two-step reaction [Rh(CH_3_COCHCNHCH_3_)(CO)(PPh_3_)] (**3A**) + CH_3_I, the lowest energy Rh(III)-acyl isomers, 13a and 14a, were ca. 0.3 eV lower in energy than the energy of the lowest energy Rh(III)-alkyl isomers, 1a and 2a, providing a driving force for CO insertion to occur in the 1a and 2a to form 13a and 14a, respectively.

The kinetic rate of the oxidative addition of methyl iodide to the two [Rh(imino-β-diketonato)(CO)(PPh)_3_] complexes, [Rh(CH_3_COCHCNHCH_3_)(CO)(PPh_3_)] **3A** and [Rh(CH_3_COCHCNPhCH_3_)(CO)(PPh_3_)] **3B**, is 7–11 times faster than the oxidative addition reaction rate of CH_3_I to [Rh(CH_3_COCHCOCH_3_)(CO)(PPh_3_)], due to the smaller electronegativity of N in the N,O-BID ligand (complexes **3A** and **3B**), compared to O in the O,O-BID ligand ([Rh(CH_3_COCHCOCH_3_)(CO)(PPh_3_)] complex). 

## Figures and Tables

**Table 3:** Observed kinetic rate constants, wavelengths, and concentrations utilized in the IR study of the oxidative addition reaction between the indicated [Rh(N,O-BID)(CO)(PPh_3_)] and CH_3_I at 25 °C.

Concentration CH_3_I (mol dm^−3^)	Reaction Step	Wavelength (cm^−1^)	k_obs_ (s^−1^)
[Rh(CH_3_COCHCNPhCH_3_)(CO)(PPh_3_)] **3B** + CH_3_I			
0.02720	Rh(I) loss	1972.62	0.00720(5)
	Rh(III)-alkyl gain	2059.72	0.007(3)
0.05383	Rh(I) loss	1972.62	0.0132(2)
	Rh(III)-alkyl gain	2059.72	0.0124(1)
0.12780	Rh(I) loss	1972.62	0.02338(4)
	Rh(III)-alkyl gain	2059.72	0.0242(2)
[Rh(CH_3_COCHCNHCH_3_)(CO)(PPh_3_)] **3A** + CH_3_I			
0.0236	Rh(I) loss	1967.66	0.00227(5)
	Rh(III)-alkyl gain	2061.30	0.0077(4)
	Rh(III)-alkyl loss	2061.30	0.00103(2)
	Rh(III)-acyl gain	1713.74	0.0014(5)
0.0537	Rh(I) loss	1967.66	0.0059(1)
	Rh(III)-alkyl gain	2061.30	0.015(1)
	Rh(III)-alkyl loss	2061.30	0.00174(3)
	Rh(III)-acyl gain	1713.74	0.00185(3)
0.1283	Rh(I) loss	1967.66	0.0158(2)
	Rh(III)-alkyl gain	2061.30	0.029(1)
	Rh(III)-alkyl loss	2061.30	0.00191(3)
	Rh(III)-acyl gain	1713.74	0.00181(2)

**Table 4:** Second-order reaction rate constants at different temperatures of the oxidative addition reaction between the [Rh(N,O-BID)(CO)(PPh_3_)] complexes and CH_3_I, as obtained from a UV–Vis kinetic study.

Complex	Temperature (K)	k_2_ (mol^−1^ dm^3^ s^−1^)
[Rh(CH_3_COCHCNHCH_3_)(CO)(PPh_3_)] **3A**	298	0.144(1)
[Rh(CH_3_COCHCNPhCH_3_)(CO)(PPh_3_)] **3B**	288	0.125(3)
	293	0.188(4)
	298	0.213(5)
	303	0.29(2)
	308	0.44(2)

**Table 5:** Pseudo first-order reaction rate constant of the methine and methyl resonance signals of the oxidative addition reaction between CH_3_I and [Rh(N,O-BID)(CO)(PPh_3_)], as obtained from an NMR kinetic study.

Reaction Step	Resonance Signal	ppm	k_obs_ (s^−1^)	Average k_obs_ (s^−1^)
[Rh(CH_3_COCHCNPhCH_3_)(CO)(PPh_3_)] **3B** + 0.0502 M CH_3_I				
Rh(I)-loss (one isomer)	Methine	5.018	0.0112(6)	0.011(1)
	O-CH_3_	1.567	0.0111(7)	
	N-CH_3_	1.688	0.0111(6)	
Rh(III)-alkyl gain (one isomer)	Methine	5.004	0.014(1)	0.014(1)
	O-CH_3_	1.655	0.014(1)	
	N-CH_3_	1.869	0.014(1)	
[Rh(CH_3_COCHCNHCH_3_)(CO)(PPh_3_)] **3A** + 0.0301 M CH_3_I				
Rh(I)-loss (two isomers)	Methine	5.002 and 4.990	0.0040(2)	0.0043(2)
	O-CH_3_	1.583 (a) and 1.838 (b)	0.0047(1)	
	N-CH_3_	2.056 (a) and 1.965 (b)	0.0042(2)	
Rh(III)-alkyl gain (one isomer)	Methine	4.881	- ^a^	- ^a^
	O-CH_3_	2.017; 1.862	- ^a^	
	N-CH_3_		- ^a^	
Rh(III)-alkyl loss (one isomer)	Methine	4.881	0.00171(4)	0.00170(4)
	O-CH_3_	2.017; 1.862	0.00170(4)	
	N-CH_3_		0.00168(4)	
Rh(III)-acyl gain (isomer A) ^b^	Methine	4.976 and 5.014	0.00157(2)	0.00152(1)
	O-CH_3_	2.157 (a) and 2.133 (b)	0.00150(1)	
	N-CH_3_	2.861 (a) and 2.555 (b)	0.00150(1)	

^a^ Unable to obtain sufficient statistical data. ^b^ Two Rh(III)-acyl isomers observed—the peaks of Rh(III)-acyl B were too small to obtain sufficient statistical data.

**Table 6:** PW91/TZP DFT-calculated energies (eV) for the reaction mechanism of the oxidative addition of CH_3_I to the applicable [Rh(N,O-BID)(CO)(PPh_3_)] complex.

Complex	Energy (eV)
[Rh(CH_3_COCHCNPhCH_3_)(CO)(PPh_3_)] **3B**	
Reactants: **3B** and CH_3_I	0.54
TS1	0.83 (imaginary frequency 270.0i cm^−1^)
Alkyl: [Rh(III)-alkyl 2a]	0.00
[Rh(CH_3_COCHCNHCH_3_)(CO)(PPh_3_)] **3A**	
Reactants: **3A** and CH_3_I	0.82
TS1	1.08 (imaginary frequency 266.0i cm^−1^)
Alkyl: [Rh(III)-alkyl product 2a]	0.26
TS2	1.17 (imaginary frequency 419.0i cm^−1^)
Acyl: [Rh(III)-acyl product 14a]	0.00

## Data Availability

Within the article and the Supplementary Materials.

## Supplementary Materials

The following supporting information can be downloaded at https://www.mdpi.com/article/10.3390/molecules27061931/s1. Optimized coordinates of the DFT calculations.

## Sample Availability

Samples of the compounds are not available from the authors.
