# Supplementary material for: Kinetic Study of the Oxidative Addition Reaction between Methyl Iodide and [Rh(imino-β-diketonato)(CO)(PPh)3] Complexes, Utilizing UV–Vis, IR Spectrophotometry, NMR Spectroscopy and DFT Calculations
Source: Molecules. 2022 Mar 16;27(6):1931. doi: 10.3390/molecules27061931 (PMC8954617; doi:10.3390/molecules27061931)
Supplement: Supplementary file 1 [file molecules-27-01931-s001.zip › molecules-1626320-supplementary.pdf]

molecules

Supplementary Material

Kinetic Study of the Oxidative Addition Reaction Between Methyl Iodide and [Rh(imino-β-diketonato)(CO)(PPh)₃] Complexes, Utilizing UV–Vis, IR Spectrophotometry, NMR Spectroscopy and DFT Calculations

Hendrik Ferreira [1], Marrigje Marianne Conradie [1,*] and Jeanet Conradie [1,2]

[1] Department of Chemistry, University of the Free State, Bloemfontein 9300, South Africa; hendrik1ferreira@gmail.com (H.F.); conradj@ufs.ac.za (J.C.)
[2] Department of Chemistry, UiT The Arctic University of Norway, N-9037 Tromsø, Norway
* Correspondence: conradiemm@ufs.ac.za

Optimized Cartesian coordinates (Å)

Table of Contents

Table of Contents 1
[Rh($CH_3COCHCNHCH_3$)(CO)(PPh_3)] and products of the oxidative addition reaction [Rh($CH_3COCHCNHCH_3$)(CO)(PPh_3)] + CH_3I 3
1. [Rh($CH_3COCHCNHCH_3$)(CO)(PPh_3)] (O-trans-CO) 3
2. [Rh($CH_3COCHCNHCH_3$)(CO)(PPh_3)] (N-trans-CO) 4
3. Alkyl 1a 5
4. Alkyl 2a 7
5. Alkyl 3a 8
6. Alkyl 4a 9
7. Alkyl 5a 11
8. Alkyl 6a 12
9. Alkyl 7a 14
10. Alkyl 8a 15
11. Alkyl 9a 16
12. Alkyl 10a 18
13. Alkyl 11a 19
14. Alkyl 12a 21
15. Acyl 13a 22
16. Acyl 14a 23
17. Acyl 16a 25

[Rh($CH_3COCHCNPhCH_3$)(CO)(PPh_3)] and products of the oxidative addition reaction [Rh($CH_3COCHCNPhCH_3$)(CO)(PPh_3)] + CH_3I 26
1. [Rh($CH_3COCHCNPhCH_3$)(CO)(PPh_3)] (O-trans-CO) 26
2. [Rh($CH_3COCHCNPhCH_3$)(CO)(PPh_3)] (N-trans-CO) 28
3. Alkyl 1a 29
4. Alkyl 2a 31
5. Alkyl 3a 32
6. Alkyl 4a 34
7. Alkyl 5a 36
8. Alkyl 6a 37
9. Alkyl 7a 39
10. Alkyl 8a 41
11. Alkyl 9a 42
12. Alkyl 10a 44
13. Alkyl 11a 45
14. Alkyl 12a 47
15. Acyl 13a 49
16. Acyl 14a 50
17. Acyl 15a 52
18. Acyl 16a 53
19. Acyl 18a 55

[Rh(CH$_3$COCHCNHCH$_3$)(CO)(PPh$_3$)] and products of the oxidative addition reaction [Rh(CH$_3$COCHCNHCH$_3$)(CO)(PPh$_3$)] + CH$_3$I

1. [Rh(CH$_3$COCHCNHCH$_3$)(CO)(PPh$_3$)] (O-trans-CO)

Rh	-1.079312000	1.014814000	1.763392000
N	-2.055962000	1.704705000	3.424024000
C	-1.771793000	1.470382000	4.691583000
C	-0.678596000	0.681904000	5.120529000
C	0.244547000	0.042070000	4.302034000
O	0.260944000	0.059844000	3.004935000
C	-2.257247000	1.867231000	0.663748000
O	-3.056966000	2.448171000	0.039748000
P	0.094543000	0.037346000	0.043899000
C	1.875995000	0.519202000	-0.099813000
C	2.535336000	1.042192000	1.022952000
C	3.875984000	1.419640000	0.937846000
C	4.570367000	1.284469000	-0.265995000
C	3.919387000	0.767175000	-1.387785000
C	2.578461000	0.387989000	-1.307308000
C	-0.510111000	0.275695000	-1.680160000
C	-0.886860000	-0.792016000	-2.504156000
C	-1.373650000	-0.549126000	-3.791405000
C	-1.483068000	0.757558000	-4.266439000
C	-1.102266000	1.827184000	-3.450548000
C	-0.622058000	1.589471000	-2.165032000
C	0.129337000	-1.796500000	0.260342000
C	1.244984000	-2.585898000	-0.045853000
C	1.207773000	-3.966328000	0.162410000
C	0.057693000	-4.569291000	0.674786000
C	-1.058021000	-3.787179000	0.983961000
C	-1.020778000	-2.407435000	0.785125000
H	1.989141000	1.146338000	1.958911000
H	4.376690000	1.829688000	1.814779000
H	5.614973000	1.589226000	-0.332537000
H	4.452278000	0.665715000	-2.332708000
H	2.073228000	0.001168000	-2.191995000
H	-0.805170000	-1.815776000	-2.140178000
H	-1.670321000	-1.388084000	-4.420278000
H	-1.868752000	0.945239000	-5.268306000
H	-1.189533000	2.850864000	-3.812783000
H	-0.339333000	2.428118000	-1.528265000

H	2.151250000	-2.122979000	-0.434506000
H	2.084746000	-4.569908000	-0.070354000
H	0.033811000	-5.646240000	0.842392000
H	-1.954642000	-4.250108000	1.394997000
H	-1.875156000	-1.785187000	1.055626000
H	-0.553146000	0.564237000	6.195456000
C	1.360688000	-0.767039000	4.919536000
H	1.322342000	-0.757870000	6.013398000
H	1.300913000	-1.803429000	4.562071000
H	2.330659000	-0.370695000	4.590087000
C	-2.651267000	2.070146000	5.766748000
H	-2.066113000	2.740534000	6.409942000
H	-3.484812000	2.640092000	5.340005000
H	-3.063153000	1.280551000	6.408720000
H	-2.880413000	2.292970000	3.310697000

2. $[Rh(CH_3COCHCNHCH_3)(CO)(PPh_3)]$ (N-trans-CO)

Rh	-1.014203000	0.761832000	1.890164000
O	-1.949348000	1.548695000	3.575167000
C	-1.373811000	2.014853000	4.629923000
C	0.000213000	2.074219000	4.868749000
C	1.010791000	1.636174000	3.992717000
N	0.779735000	1.125829000	2.791513000
C	-2.681907000	0.335619000	1.197207000
O	-3.741616000	0.049524000	0.806866000
P	-0.037931000	-0.127727000	0.072727000
C	1.812114000	-0.028733000	0.020498000
C	2.398265000	1.249061000	-0.016556000
C	3.785628000	1.390992000	-0.033162000
C	4.607112000	0.260911000	-0.009881000
C	4.033755000	-1.010476000	0.028579000
C	2.643278000	-1.156864000	0.045407000
C	-0.462966000	0.614370000	-1.564899000
C	0.217385000	0.247081000	-2.737643000
C	-0.100144000	0.849272000	-3.953891000
C	-1.089895000	1.834452000	-4.012394000
C	-1.757906000	2.217283000	-2.849202000
C	-1.444973000	1.611662000	-1.630303000
C	-0.371284000	-1.938181000	-0.087112000
C	-0.275399000	-2.696719000	1.092687000
C	-0.530938000	-4.066760000	1.075474000
C	-0.913804000	-4.692686000	-0.114296000

C	-1.034336000	-3.941663000	-1.283429000
C	-0.760304000	-2.571418000	-1.273622000
H	1.761731000	2.134516000	-0.029229000
H	4.225426000	2.387084000	-0.063980000
H	5.691451000	0.372426000	-0.021757000
H	4.667219000	-1.897058000	0.045357000
H	2.205955000	-2.154542000	0.074557000
H	1.008563000	-0.500899000	-2.696448000
H	0.433744000	0.556818000	-4.857427000
H	-1.331043000	2.309517000	-4.963547000
H	-2.520755000	2.994173000	-2.884261000
H	-1.944429000	1.924280000	-0.713654000
H	-0.023977000	-2.195752000	2.028419000
H	-0.452053000	-4.642270000	1.997316000
H	-1.133216000	-5.760133000	-0.124725000
H	-1.353010000	-4.418676000	-2.209734000
H	-0.871704000	-1.995885000	-2.190970000
H	0.321133000	2.492831000	5.820793000
C	2.443643000	1.724440000	4.473156000
H	2.690763000	0.838258000	5.075030000
H	3.146647000	1.767964000	3.632541000
H	2.594567000	2.605628000	5.106581000
C	-2.330047000	2.534968000	5.680908000
H	-2.935377000	3.346358000	5.255894000
H	-3.021979000	1.735438000	5.975778000
H	-1.806962000	2.905058000	6.568597000
H	1.641620000	0.873059000	2.304375000

3. Alkyl 1a

Rh	-0.897145000	0.652971000	2.004978000
O	-1.739591000	1.498345000	3.739287000
C	-1.072779000	2.147855000	4.642107000
C	0.294636000	2.402579000	4.660240000
C	1.248207000	1.978325000	3.702851000
N	0.952953000	1.296460000	2.619059000
C	-2.642816000	0.131467000	1.557972000
O	-3.746662000	-0.144525000	1.347550000
P	-0.001650000	-0.217132000	0.019868000
C	1.825682000	-0.049037000	0.003431000

C	2.433936000	1.141881000	-0.428523000
C	3.818253000	1.297603000	-0.338484000
C	4.605636000	0.275236000	0.197040000
C	4.003946000	-0.900603000	0.653842000
C	2.620563000	-1.062566000	0.564719000
C	-0.561137000	0.607956000	-1.539493000
C	0.247853000	0.613675000	-2.688179000
C	-0.228560000	1.149910000	-3.885626000
C	-1.521065000	1.675850000	-3.956643000
C	-2.336914000	1.662103000	-2.823553000
C	-1.859311000	1.133239000	-1.623238000
C	-0.298465000	-1.995636000	-0.381931000
C	-1.357163000	-2.707389000	0.197236000
C	-1.622833000	-4.021130000	-0.197178000
C	-0.829543000	-4.637413000	-1.166490000
C	0.230100000	-3.933905000	-1.747289000
C	0.491241000	-2.618914000	-1.363196000
H	1.830869000	1.949120000	-0.840810000
H	4.278517000	2.221075000	-0.686059000
H	5.686079000	0.397201000	0.264258000
H	4.610321000	-1.695703000	1.084262000
H	2.157350000	-1.974533000	0.938860000
H	1.255019000	0.203154000	-2.656454000
H	0.414198000	1.152053000	-4.764523000
H	-1.890988000	2.093856000	-4.892038000
H	-3.346768000	2.066566000	-2.868445000
H	-2.506476000	1.139972000	-0.750195000
H	-1.958822000	-2.257658000	0.982021000
H	-2.445292000	-4.563860000	0.266135000
H	-1.033140000	-5.664490000	-1.467924000
H	0.855582000	-4.406830000	-2.502884000
H	1.317128000	-2.084329000	-1.829339000
H	0.676758000	2.970789000	5.506023000
C	2.696352000	2.314010000	3.973476000
H	2.802556000	3.371485000	4.243469000
H	3.060304000	1.722236000	4.824088000
H	3.333311000	2.102954000	3.106700000
C	-1.932004000	2.651978000	5.777554000
H	-2.741654000	3.278873000	5.382515000
H	-2.398538000	1.798599000	6.287013000
H	-1.348796000	3.226804000	6.502946000
H	1.763744000	1.061093000	2.043393000
C	-1.102170000	2.593071000	1.189663000
H	-0.856002000	3.248270000	2.032109000

H	-0.399085000	2.749492000	0.364877000
H	-2.127841000	2.775705000	0.852558000
I	-0.463129000	-1.743233000	3.575840000

4. Alkyl 2a

Rh	-1.234018000	-0.812886000	1.833728000
N	-2.096128000	-1.579039000	3.545740000
C	-1.506003000	-1.806026000	4.695652000
C	-0.135602000	-1.544676000	4.950013000
C	0.796121000	-1.050686000	4.045589000
O	0.585509000	-0.734457000	2.802780000
C	-2.863922000	-0.750743000	0.962326000
O	-3.919636000	-0.679714000	0.490103000
P	-0.052238000	0.129262000	-0.026395000
C	1.759279000	-0.238125000	-0.074602000
C	2.242749000	-1.457411000	0.422014000
C	3.599428000	-1.769506000	0.319852000
C	4.487835000	-0.870345000	-0.274848000
C	4.011282000	0.343851000	-0.776701000
C	2.655222000	0.658816000	-0.679822000
C	-0.631477000	-0.304077000	-1.726487000
C	-0.149885000	0.422285000	-2.829676000
C	-0.537048000	0.077641000	-4.123495000
C	-1.405830000	-0.998462000	-4.334001000
C	-1.881097000	-1.728988000	-3.244275000
C	-1.494288000	-1.386076000	-1.945592000
C	-0.131441000	1.966016000	0.001252000
C	0.729670000	2.688923000	0.842800000
C	0.602205000	4.073233000	0.960235000
C	-0.396233000	4.749661000	0.253754000
C	-1.271531000	4.033858000	-0.566784000
C	-1.143161000	2.648851000	-0.690837000
H	1.557998000	-2.164663000	0.885711000
H	3.961519000	-2.719923000	0.710596000
H	5.547387000	-1.114163000	-0.348890000
H	4.693698000	1.051095000	-1.245947000
H	2.304540000	1.611574000	-1.071817000
H	0.524894000	1.263563000	-2.682900000
H	-0.157301000	0.650112000	-4.968436000
H	-1.705116000	-1.268243000	-5.346315000
H	-2.548923000	-2.575178000	-3.399142000
H	-1.837731000	-1.990383000	-1.107457000

H	1.503518000	2.169421000	1.406588000
H	1.283192000	4.623228000	1.608040000
H	-0.494236000	5.830776000	0.346107000
H	-2.057154000	4.551463000	-1.115307000
H	-1.832208000	2.103033000	-1.334405000
H	0.221136000	-1.763739000	5.954546000
C	2.223220000	-0.835716000	4.486881000
H	2.379907000	-1.141507000	5.525370000
H	2.485679000	0.225303000	4.379348000
H	2.899521000	-1.402501000	3.833651000
C	-2.319585000	-2.392757000	5.823818000
H	-1.899905000	-3.361974000	6.122320000
H	-3.367355000	-2.535000000	5.538429000
H	-2.276262000	-1.735626000	6.701516000
H	-3.082118000	-1.836382000	3.525002000
C	-1.614604000	1.088783000	2.660256000
H	-2.015388000	1.788646000	1.919213000
H	-0.641635000	1.428983000	3.031306000
H	-2.321524000	0.940383000	3.483691000
I	-0.875756000	-3.635688000	1.080137000

5. Alkyl 3a

Rh	-0.929560000	-0.726819000	1.929039000
N	-1.701137000	-1.566527000	3.622814000
C	-1.079430000	-2.351359000	4.474123000
C	0.302805000	-2.654577000	4.410787000
C	1.209616000	-2.202723000	3.454245000
O	0.952081000	-1.432249000	2.443606000
C	-2.576511000	-0.233097000	1.262916000
O	-3.627708000	-0.010511000	0.826830000
P	-0.392548000	1.499603000	3.082222000
C	-1.084863000	1.694801000	4.786169000
C	-0.689381000	0.758079000	5.756626000
C	-1.178678000	0.837671000	7.060496000
C	-2.079694000	1.847785000	7.413256000
C	-2.476170000	2.783276000	6.455753000
C	-1.979960000	2.710668000	5.150668000
C	1.393917000	1.882470000	3.357662000
C	1.788346000	2.758208000	4.383272000
C	3.138817000	3.043883000	4.588108000
C	4.111311000	2.457114000	3.772534000
C	3.725167000	1.590219000	2.748027000

C	2.373976000	1.301922000	2.539542000
C	-1.034969000	2.965497000	2.170253000
C	-2.411492000	3.053390000	1.903765000
C	-2.924855000	4.122416000	1.169590000
C	-2.066948000	5.111367000	0.677960000
C	-0.697024000	5.026527000	0.931786000
C	-0.182702000	3.961061000	1.675375000
H	0.015669000	-0.032530000	5.495827000
H	-0.856299000	0.108680000	7.803275000
H	-2.466999000	1.906551000	8.430087000
H	-3.169007000	3.580532000	6.721917000
H	-2.288933000	3.457588000	4.421595000
H	1.039512000	3.217688000	5.027707000
H	3.429776000	3.724150000	5.387653000
H	5.166321000	2.674710000	3.937909000
H	4.477601000	1.128751000	2.109414000
H	2.082221000	0.618715000	1.745825000
H	-3.094091000	2.290538000	2.275550000
H	-3.995207000	4.178852000	0.976685000
H	-2.466002000	5.941779000	0.096502000
H	-0.019972000	5.789428000	0.550117000
H	0.887999000	3.905328000	1.865501000
H	0.690706000	-3.308242000	5.189494000
C	2.656667000	-2.619710000	3.549632000
H	2.843706000	-3.251789000	4.422581000
H	3.292107000	-1.725694000	3.604383000
H	2.942516000	-3.164442000	2.640237000
C	-1.874592000	-2.949361000	5.609826000
H	-2.934591000	-2.679749000	5.550842000
H	-1.476867000	-2.602630000	6.572249000
H	-1.786245000	-4.042895000	5.595894000
H	-2.698343000	-1.455721000	3.802882000
C	-1.216147000	-2.605806000	0.989358000
H	-1.916932000	-3.201131000	1.586634000
H	-0.216241000	-3.053002000	0.993060000
H	-1.576866000	-2.490217000	-0.038011000
I	0.129277000	0.183446000	-0.455607000

6. Alkyl 4a

Rh	-1.047328000	0.653055000	1.873997000
O	-2.113526000	1.370652000	3.559459000
C	-1.567871000	1.607786000	4.708301000

C	-0.223761000	1.469829000	5.052867000
C	0.834057000	1.036859000	4.217183000
N	0.699166000	0.701234000	2.953620000
C	-0.961732000	2.536103000	1.292606000
O	-1.026564000	3.671984000	1.097194000
P	0.016292000	-0.289843000	0.029453000
C	1.830146000	0.101586000	-0.037609000
C	2.261375000	1.386051000	0.337462000
C	3.600587000	1.757252000	0.200365000
C	4.528486000	0.851591000	-0.318385000
C	4.108302000	-0.422991000	-0.706709000
C	2.769757000	-0.795756000	-0.572168000
C	-0.497337000	0.238623000	-1.667833000
C	-0.009591000	-0.469745000	-2.778857000
C	-0.308185000	-0.043587000	-4.072968000
C	-1.085075000	1.101858000	-4.273455000
C	-1.564327000	1.815532000	-3.173841000
C	-1.274784000	1.384628000	-1.876752000
C	-0.066211000	-2.120903000	-0.019278000
C	0.801311000	-2.892335000	0.770443000
C	0.673242000	-4.280610000	0.808935000
C	-0.335668000	-4.912197000	0.076777000
C	-1.220575000	-4.148064000	-0.688014000
C	-1.090729000	-2.758853000	-0.734486000
H	1.549670000	2.105122000	0.738966000
H	3.915182000	2.756348000	0.498005000
H	5.573793000	1.139704000	-0.424281000
H	4.822478000	-1.133097000	-1.121199000
H	2.462112000	-1.789559000	-0.890113000
H	0.601816000	-1.359660000	-2.639558000
H	0.070598000	-0.606744000	-4.924442000
H	-1.315763000	1.435607000	-5.284603000
H	-2.173325000	2.706296000	-3.319998000
H	-1.682268000	1.931251000	-1.031002000
H	1.584202000	-2.414291000	1.356857000
H	1.360620000	-4.867485000	1.416348000
H	-0.435263000	-5.996654000	0.107801000
H	-2.017414000	-4.631219000	-1.251228000
H	-1.792266000	-2.171516000	-1.324790000
H	0.045780000	1.717151000	6.077880000
C	2.212757000	0.955357000	4.832757000
H	2.218778000	0.208735000	5.637351000
H	2.975075000	0.680111000	4.095235000
H	2.485713000	1.918714000	5.280539000

C	-2.550054000	2.073942000	5.758521000
H	-3.079690000	2.965734000	5.399907000
H	-3.304413000	1.293454000	5.923970000
H	-2.055211000	2.302359000	6.706954000
H	1.567539000	0.424884000	2.493448000
C	-1.345028000	-1.245051000	2.750636000
H	-2.008836000	-1.047077000	3.601199000
H	-1.824258000	-1.939918000	2.052409000
H	-0.385429000	-1.649377000	3.099030000
I	-3.547566000	0.328965000	0.745663000

7. Alkyl 5a

Rh	-0.930377000	-0.618353000	1.861925000
O	-1.726201000	-1.620929000	3.506372000
C	-1.003604000	-2.195502000	4.423114000
C	0.380517000	-2.312855000	4.469562000
C	1.310944000	-1.825804000	3.514205000
N	0.958006000	-1.185108000	2.425980000
C	-2.615068000	0.072607000	1.424496000
O	-3.641607000	0.558889000	1.201105000
P	-1.085252000	-2.689906000	0.400629000
C	0.560308000	-3.364646000	-0.065584000
C	1.335156000	-4.052912000	0.883611000
C	2.624118000	-4.483179000	0.566753000
C	3.164973000	-4.215627000	-0.694568000
C	2.409038000	-3.513365000	-1.636249000
C	1.115276000	-3.088225000	-1.324443000
C	-1.966937000	-4.111700000	1.184774000
C	-1.570771000	-5.448746000	1.028506000
C	-2.312345000	-6.479379000	1.609645000
C	-3.461794000	-6.188612000	2.348869000
C	-3.869189000	-4.860582000	2.501233000
C	-3.125775000	-3.828259000	1.926194000
C	-1.964615000	-2.564253000	-1.224281000
C	-2.418314000	-1.328570000	-1.702813000
C	-3.109673000	-1.248048000	-2.915466000
C	-3.351582000	-2.402274000	-3.661239000
C	-2.895513000	-3.639760000	-3.194029000
C	-2.208254000	-3.721009000	-1.983701000
H	0.927195000	-4.263829000	1.871851000
H	3.207525000	-5.027832000	1.308232000
H	4.172019000	-4.551177000	-0.940640000

H	2.823048000	-3.294895000	-2.619737000
H	0.536168000	-2.541414000	-2.067221000
H	-0.679541000	-5.693476000	0.453101000
H	-1.989632000	-7.511809000	1.480088000
H	-4.037476000	-6.993807000	2.804529000
H	-4.764495000	-4.625018000	3.075459000
H	-3.442177000	-2.797339000	2.068873000
H	-2.212055000	-0.418668000	-1.143522000
H	-3.457137000	-0.279902000	-3.273303000
H	-3.895028000	-2.341198000	-4.603612000
H	-3.079696000	-4.545352000	-3.770235000
H	-1.866128000	-4.692019000	-1.628006000
H	0.796926000	-2.830104000	5.331505000
C	2.775908000	-2.075781000	3.779986000
H	3.074261000	-1.592966000	4.719532000
H	3.405867000	-1.692890000	2.970434000
H	2.962305000	-3.150956000	3.896586000
C	-1.832777000	-2.784557000	5.540040000
H	-2.536202000	-3.519239000	5.126002000
H	-2.427207000	-1.994208000	6.016872000
H	-1.206294000	-3.269298000	6.294608000
H	1.728493000	-0.890427000	1.822526000
C	-0.693927000	1.040258000	3.163617000
H	0.378261000	1.136742000	3.364350000
H	-1.244951000	0.789474000	4.078383000
H	-1.077550000	1.965245000	2.719155000
I	0.236259000	0.891481000	-0.095906000

8. Alkyl 6a

Rh	-1.028139000	-0.591391000	1.840656000
N	-2.105585000	-1.302733000	3.459382000
C	-1.626033000	-1.768079000	4.582338000
C	-0.233521000	-1.852982000	4.873053000
C	0.817800000	-1.415994000	4.082977000
O	0.758964000	-0.875822000	2.896552000
C	-1.081135000	-2.374283000	0.998794000
O	-1.294924000	-3.393174000	0.499546000
P	0.283522000	0.370473000	0.108748000
C	1.378095000	1.793685000	0.571700000
C	1.906586000	1.862589000	1.870432000
C	2.805693000	2.874803000	2.212843000
C	3.189295000	3.826604000	1.264959000

C	2.676720000	3.756015000	-0.033115000
C	1.780220000	2.743761000	-0.381330000
C	1.517263000	-0.802023000	-0.621439000
C	2.094100000	-0.556519000	-1.878501000
C	3.066861000	-1.417472000	-2.387449000
C	3.486843000	-2.523958000	-1.642569000
C	2.932859000	-2.762252000	-0.383433000
C	1.953241000	-1.905937000	0.126221000
C	-0.683176000	0.983034000	-1.323991000
C	-1.236059000	2.272252000	-1.296258000
C	-2.068806000	2.704133000	-2.328439000
C	-2.370479000	1.849418000	-3.391705000
C	-1.841523000	0.556688000	-3.413537000
C	-1.007021000	0.122713000	-2.382940000
H	1.620174000	1.113613000	2.604687000
H	3.206353000	2.918877000	3.225198000
H	3.887281000	4.618350000	1.535229000
H	2.974165000	4.489256000	-0.781533000
H	1.394457000	2.703339000	-1.398198000
H	1.780522000	0.302490000	-2.469148000
H	3.498298000	-1.221158000	-3.368034000
H	4.247176000	-3.194346000	-2.041768000
H	3.262439000	-3.615941000	0.207534000
H	1.544846000	-2.079039000	1.119844000
H	-1.019726000	2.944916000	-0.467788000
H	-2.486220000	3.709494000	-2.296645000
H	-3.021412000	2.187702000	-4.197316000
H	-2.080125000	-0.120430000	-4.232435000
H	-0.609393000	-0.890968000	-2.408215000
H	0.029845000	-2.274655000	5.841229000
C	2.233802000	-1.538105000	4.595969000
H	2.267928000	-2.012339000	5.580957000
H	2.692067000	-0.542197000	4.661435000
H	2.836064000	-2.124959000	3.890256000
C	-2.581981000	-2.244669000	5.650018000
H	-2.375540000	-3.294310000	5.895344000
H	-3.625250000	-2.153717000	5.329979000
H	-2.439243000	-1.663649000	6.570129000
H	-3.125121000	-1.280814000	3.386569000
C	-1.232524000	1.296120000	2.771242000
H	-2.231589000	1.341664000	3.220383000
H	-1.110749000	2.133889000	2.073435000
H	-0.465268000	1.351815000	3.555594000
I	-3.472353000	-0.133939000	0.693703000

9. Alkyl 7a

Rh	-0.861044000	0.736940000	1.983159000
N	-1.804962000	1.550580000	3.633213000
C	-1.244340000	1.983887000	4.738761000
C	0.147899000	1.940216000	5.012759000
C	1.163969000	1.421184000	4.211440000
O	1.044218000	0.903193000	3.030624000
C	-0.846531000	2.401244000	1.167099000
O	-0.973636000	3.435540000	0.662434000
P	0.283601000	-0.264519000	0.155697000
C	1.399356000	-1.698732000	0.491351000
C	2.027023000	-1.812967000	1.740456000
C	2.957080000	-2.830177000	1.963554000
C	3.267109000	-3.738929000	0.948332000
C	2.650237000	-3.622311000	-0.299878000
C	1.724347000	-2.602824000	-0.532470000
C	1.462318000	0.905818000	-0.659802000
C	1.822527000	0.780380000	-2.011008000
C	2.778579000	1.633732000	-2.564365000
C	3.396926000	2.606461000	-1.773470000
C	3.059762000	2.719585000	-0.422644000
C	2.098308000	1.873226000	0.134114000
C	-0.857498000	-0.831582000	-1.158595000
C	-1.412006000	-2.119171000	-1.086556000
C	-2.384698000	-2.517489000	-2.003817000
C	-2.821198000	-1.633823000	-2.994057000
C	-2.289457000	-0.343144000	-3.056533000
C	-1.318943000	0.060680000	-2.137990000
H	1.790003000	-1.101708000	2.528716000
H	3.438674000	-2.913746000	2.937318000
H	3.988553000	-4.534846000	1.127611000
H	2.889646000	-4.322708000	-1.098978000
H	1.257887000	-2.520026000	-1.512754000
H	1.357993000	0.021041000	-2.638350000
H	3.042226000	1.534321000	-3.616237000
H	4.143568000	3.269895000	-2.208434000
H	3.548273000	3.465689000	0.203045000
H	1.858702000	1.944104000	1.194911000
H	-1.089587000	-2.809532000	-0.308403000
H	-2.804303000	-3.520343000	-1.939176000
H	-3.579809000	-1.947426000	-3.710325000

H	-2.631201000	0.355391000	-3.818644000
H	-0.922234000	1.073742000	-2.190788000
H	0.450419000	2.339384000	5.979440000
C	2.582517000	1.427406000	4.734801000
H	2.665805000	1.928555000	5.703615000
H	2.936867000	0.392665000	4.837200000
H	3.241752000	1.922887000	4.010723000
C	-2.137476000	2.552210000	5.817200000
H	-3.183428000	2.597240000	5.495515000
H	-2.072607000	1.933103000	6.721524000
H	-1.804914000	3.561424000	6.089911000
H	-2.821009000	1.643179000	3.602959000
C	-2.770618000	0.502118000	1.093768000
H	-3.518612000	0.775613000	1.850466000
H	-2.901897000	1.133344000	0.205179000
H	-2.894533000	-0.550713000	0.820649000
I	-1.320219000	-1.734701000	3.163761000

10. Alkyl 8a

Rh	-0.767726000	0.782061000	2.005460000
O	-1.728092000	1.537750000	3.727824000
C	-1.110367000	1.874718000	4.822607000
C	0.249333000	1.806632000	5.099372000
C	1.289804000	1.320209000	4.257335000
N	1.101884000	0.889101000	3.035655000
C	-0.612903000	2.532195000	1.410124000
O	-0.513348000	3.656355000	1.154863000
P	0.147506000	-0.150262000	0.069373000
C	1.927856000	-0.518400000	0.334706000
C	2.881086000	0.510679000	0.249695000
C	4.218496000	0.260436000	0.562528000
C	4.615446000	-1.014044000	0.976408000
C	3.667970000	-2.034926000	1.086530000
C	2.329674000	-1.789411000	0.774831000
C	0.127330000	0.964851000	-1.409306000
C	1.122961000	0.883563000	-2.397028000
C	1.049800000	1.679175000	-3.541125000
C	-0.022752000	2.556465000	-3.721934000
C	-1.025675000	2.631728000	-2.753614000
C	-0.952053000	1.840305000	-1.605437000
C	-0.542168000	-1.712209000	-0.639837000
C	-1.825266000	-2.165766000	-0.305773000

C	-2.353184000	-3.300733000	-0.926085000
C	-1.604400000	-3.995201000	-1.877995000
C	-0.323290000	-3.547990000	-2.214969000
C	0.203647000	-2.409232000	-1.605145000
H	2.583721000	1.510133000	-0.065315000
H	4.948335000	1.064653000	0.483015000
H	5.659605000	-1.209137000	1.218212000
H	3.967423000	-3.027402000	1.419766000
H	1.595738000	-2.586414000	0.880593000
H	1.966677000	0.207058000	-2.275881000
H	1.835794000	1.611237000	-4.291686000
H	-0.076743000	3.177504000	-4.615311000
H	-1.869002000	3.307503000	-2.887106000
H	-1.745718000	1.905836000	-0.864531000
H	-2.406838000	-1.646759000	0.449451000
H	-3.350413000	-3.644267000	-0.654693000
H	-2.015813000	-4.883611000	-2.355940000
H	0.269678000	-4.083970000	-2.954412000
H	1.201913000	-2.074199000	-1.880688000
H	0.556161000	2.142930000	6.088579000
C	2.681466000	1.288746000	4.847793000
H	2.710422000	0.598814000	5.701584000
H	3.427508000	0.966671000	4.112595000
H	2.960485000	2.280776000	5.224680000
C	-2.049388000	2.387680000	5.891199000
H	-2.616322000	3.245863000	5.507728000
H	-2.775738000	1.604717000	6.146122000
H	-1.511046000	2.685388000	6.795878000
H	1.952995000	0.551378000	2.580880000
C	-2.775186000	0.697608000	1.289445000
H	-3.256089000	-0.106065000	1.858618000
H	-3.242122000	1.654414000	1.555429000
H	-2.902044000	0.516459000	0.216227000
I	-1.009714000	-1.734878000	3.167396000

11. Alkyl 9a

Rh	-0.844484000	0.757603000	2.085198000
N	-2.317230000	1.498003000	3.446878000
C	-2.211378000	2.550334000	4.219642000
C	-0.999381000	3.281984000	4.385671000
C	0.236789000	2.999801000	3.821984000
O	0.532679000	2.027311000	3.004543000

C	-1.990493000	-0.234380000	1.031785000
O	-2.712962000	-0.802090000	0.326040000
P	-0.175787000	-1.094464000	3.384196000
C	-0.697235000	-1.132591000	5.154466000
C	-0.653462000	0.057518000	5.895050000
C	-0.940617000	0.047210000	7.261674000
C	-1.272638000	-1.149288000	7.901359000
C	-1.305745000	-2.339997000	7.170761000
C	-1.014856000	-2.335934000	5.805413000
C	1.640022000	-1.392762000	3.532191000
C	2.113791000	-2.665533000	3.892849000
C	3.476849000	-2.871875000	4.104027000
C	4.377138000	-1.810457000	3.970177000
C	3.907869000	-0.540961000	3.626997000
C	2.544650000	-0.327854000	3.407994000
C	-0.871243000	-2.624789000	2.650948000
C	-2.206602000	-2.970152000	2.919242000
C	-2.801630000	-4.046112000	2.262604000
C	-2.076795000	-4.780460000	1.319595000
C	-0.754533000	-4.432174000	1.035464000
C	-0.152844000	-3.359041000	1.695533000
H	-0.394873000	0.998180000	5.412045000
H	-0.904686000	0.978951000	7.825458000
H	-1.501166000	-1.155391000	8.966668000
H	-1.555355000	-3.279129000	7.662295000
H	-1.035542000	-3.274365000	5.255030000
H	1.423413000	-3.499290000	4.009556000
H	3.833803000	-3.863542000	4.377949000
H	5.441219000	-1.973478000	4.138404000
H	4.604045000	0.291188000	3.528641000
H	2.178287000	0.664658000	3.152527000
H	-2.787344000	-2.395786000	3.640006000
H	-3.835227000	-4.306376000	2.484618000
H	-2.544288000	-5.618257000	0.803535000
H	-0.185246000	-4.994086000	0.296750000
H	0.878142000	-3.098464000	1.463181000
H	-1.042843000	4.141130000	5.052842000
C	1.422666000	3.869366000	4.162875000
H	1.158306000	4.665331000	4.865124000
H	2.222439000	3.255380000	4.598875000
H	1.823023000	4.316554000	3.243452000
C	-3.410730000	3.029710000	5.001263000
H	-3.640455000	4.070102000	4.736370000
H	-4.294940000	2.413781000	4.804274000

H	-3.198766000	3.010415000	6.078296000
H	-3.254980000	1.095565000	3.447885000
C	0.711071000	0.293218000	0.707886000
H	1.105313000	-0.725852000	0.807129000
H	0.363191000	0.449178000	-0.319753000
H	1.493420000	1.023289000	0.947799000
I	-1.554082000	2.856298000	0.365777000

12. Alkyl 10a

Rh	-0.849479000	0.801800000	2.108368000
O	-2.778119000	1.299848000	2.965506000
C	-2.937138000	2.022356000	4.021523000
C	-1.935890000	2.573354000	4.827733000
C	-0.537091000	2.453650000	4.656374000
N	0.038109000	1.772967000	3.686280000
C	-1.634696000	0.096303000	0.561510000
O	-2.131015000	-0.287573000	-0.411601000
P	-0.683641000	-1.263753000	3.200754000
C	0.654245000	-1.318687000	4.455559000
C	1.873905000	-1.950908000	4.170547000
C	2.917509000	-1.920228000	5.098905000
C	2.752176000	-1.261581000	6.318801000
C	1.539947000	-0.626402000	6.607690000
C	0.499268000	-0.644730000	5.679762000
C	-0.331783000	-2.719236000	2.117748000
C	-0.521953000	-4.010626000	2.638720000
C	-0.273218000	-5.132902000	1.849714000
C	0.166219000	-4.982039000	0.530977000
C	0.365372000	-3.703409000	0.009310000
C	0.120691000	-2.577318000	0.799164000
C	-2.214100000	-1.771267000	4.087897000
C	-3.451698000	-1.561959000	3.457673000
C	-4.631527000	-1.989290000	4.067617000
C	-4.590657000	-2.625469000	5.311435000
C	-3.362513000	-2.841393000	5.940552000
C	-2.178668000	-2.419185000	5.332592000
H	2.010989000	-2.477618000	3.227102000
H	3.857600000	-2.417507000	4.865543000
H	3.564766000	-1.242808000	7.044347000
H	1.404154000	-0.112782000	7.558521000
H	-0.439204000	-0.143302000	5.913375000
H	-0.874366000	-4.143814000	3.660505000

H	-0.431460000	-6.126954000	2.265013000
H	0.350336000	-5.859783000	-0.087346000
H	0.709307000	-3.574987000	-1.015653000
H	0.291402000	-1.589482000	0.381939000
H	-3.499926000	-1.049367000	2.500466000
H	-5.585853000	-1.817749000	3.571569000
H	-5.513840000	-2.952135000	5.789240000
H	-3.319903000	-3.342122000	6.906844000
H	-1.229121000	-2.601026000	5.832058000
H	-2.264932000	3.165842000	5.679886000
C	0.344403000	3.153270000	5.664382000
H	0.122796000	2.794734000	6.677749000
H	1.407687000	2.990528000	5.458117000
H	0.145502000	4.232507000	5.652935000
C	-4.382698000	2.279795000	4.385335000
H	-4.880284000	2.801916000	3.557405000
H	-4.901035000	1.321586000	4.523656000
H	-4.479768000	2.878338000	5.296208000
H	1.058303000	1.779877000	3.715733000
C	1.120036000	0.573830000	1.353333000
H	1.648689000	-0.270257000	1.813816000
H	1.135061000	0.466406000	0.261593000
H	1.629629000	1.509867000	1.611930000
I	-0.963953000	3.235011000	0.722838000

13. Alkyl 11a

Rh	-1.121475000	0.444725000	1.630138000
N	-0.836280000	1.595277000	3.391207000
C	0.314896000	1.958295000	3.893114000
C	1.561640000	1.698251000	3.250739000
C	1.780497000	1.084267000	2.024197000
O	0.906528000	0.582683000	1.194858000
C	-0.863184000	-1.217274000	2.474012000
O	-0.726766000	-2.265411000	2.940100000
P	-1.609162000	2.490901000	0.376921000
C	-2.940959000	2.452478000	-0.910888000
C	-3.747227000	1.324915000	-1.112826000
C	-4.736261000	1.331617000	-2.100473000
C	-4.930013000	2.463312000	-2.893817000
C	-4.131303000	3.594223000	-2.695997000
C	-3.142008000	3.588739000	-1.713723000
C	-0.170731000	3.147992000	-0.581127000

C	0.086827000	4.521268000	-0.713675000
C	1.156570000	4.969091000	-1.491473000
C	1.979077000	4.051803000	-2.150821000
C	1.722471000	2.683392000	-2.032825000
C	0.656694000	2.231913000	-1.252624000
C	-2.119565000	3.885638000	1.463036000
C	-3.474508000	4.214742000	1.614203000
C	-3.862115000	5.219747000	2.504988000
C	-2.902572000	5.902515000	3.255370000
C	-1.551027000	5.569781000	3.120024000
C	-1.161973000	4.561356000	2.238090000
H	-3.613805000	0.443983000	-0.491458000
H	-5.354056000	0.446502000	-2.246555000
H	-5.698392000	2.465478000	-3.666348000
H	-4.274590000	4.481986000	-3.310109000
H	-2.523908000	4.474701000	-1.575723000
H	-0.542499000	5.250649000	-0.207111000
H	1.343483000	6.038440000	-1.581149000
H	2.815596000	4.402373000	-2.754624000
H	2.358188000	1.960831000	-2.543614000
H	0.479475000	1.164651000	-1.155115000
H	-4.231798000	3.689681000	1.033775000
H	-4.917498000	5.467842000	2.607656000
H	-3.205572000	6.690509000	3.944503000
H	-0.796147000	6.095849000	3.703167000
H	-0.106416000	4.307879000	2.145174000
H	2.450777000	2.040987000	3.777610000
C	3.196937000	0.945519000	1.514808000
H	3.920573000	1.381704000	2.209639000
H	3.434063000	-0.115952000	1.365105000
H	3.285988000	1.440698000	0.538773000
C	0.362443000	2.708596000	5.202911000
H	0.795783000	3.706272000	5.052816000
H	-0.635494000	2.820064000	5.639892000
H	1.007058000	2.182666000	5.918643000
H	-1.649866000	1.860185000	3.951466000
C	-1.248999000	-0.689371000	-0.174105000
H	-2.044407000	-1.445428000	-0.124766000
H	-1.430559000	-0.051709000	-1.045723000
H	-0.275204000	-1.184525000	-0.293234000
I	-3.750024000	0.139715000	2.360851000

14. Alkyl 12a

Rh	-1.070732000	0.704095000	2.009624000
N	-0.778265000	1.326525000	3.925490000
C	0.235617000	2.033739000	4.381311000
C	1.388381000	2.368834000	3.633882000
C	1.690686000	2.001115000	2.315300000
O	0.950072000	1.325212000	1.506924000
C	-1.811363000	2.357645000	1.503339000
O	-2.315404000	3.351183000	1.199082000
P	-0.307331000	-1.547516000	2.623952000
C	-1.165131000	-3.006240000	1.875163000
C	-2.384004000	-2.875007000	1.197598000
C	-3.020492000	-4.000014000	0.666423000
C	-2.445681000	-5.264089000	0.805990000
C	-1.230250000	-5.402833000	1.483295000
C	-0.593282000	-4.281644000	2.014441000
C	-0.504658000	-1.854217000	4.429909000
C	-1.584331000	-2.608395000	4.914009000
C	-1.789479000	-2.750402000	6.289376000
C	-0.916130000	-2.145924000	7.195377000
C	0.162126000	-1.392101000	6.721041000
C	0.361850000	-1.237421000	5.349079000
C	1.470704000	-1.888116000	2.277231000
C	2.004717000	-1.439460000	1.057878000
C	3.333501000	-1.715342000	0.730671000
C	4.142753000	-2.435325000	1.613850000
C	3.614698000	-2.889602000	2.824920000
C	2.285254000	-2.619836000	3.155325000
H	-2.829257000	-1.894062000	1.065022000
H	-3.963422000	-3.882034000	0.134426000
H	-2.938752000	-6.139145000	0.383770000
H	-0.772010000	-6.384546000	1.594142000
H	0.355906000	-4.403633000	2.533967000
H	-2.265889000	-3.096762000	4.218646000
H	-2.631482000	-3.340892000	6.647467000
H	-1.072806000	-2.262242000	8.267350000
H	0.849841000	-0.920019000	7.421582000
H	1.202768000	-0.643294000	4.992695000
H	1.384867000	-0.866148000	0.370899000
H	3.738555000	-1.360034000	-0.216003000
H	5.181855000	-2.642816000	1.359229000
H	4.235012000	-3.457725000	3.516903000
H	1.887550000	-2.982726000	4.101409000

H	2.140065000	2.955107000	4.159862000
C	3.026578000	2.414684000	1.738188000
H	3.619261000	3.004223000	2.444137000
H	3.590713000	1.515939000	1.454393000
H	2.866425000	2.998398000	0.822529000
C	0.178744000	2.504221000	5.817001000
H	0.249548000	3.598585000	5.854565000
H	-0.748625000	2.194899000	6.310696000
H	1.030107000	2.103675000	6.381454000
H	-1.535046000	1.165343000	4.591394000
C	-3.021829000	0.139713000	2.603296000
H	-3.001215000	-0.800111000	3.167829000
H	-3.422076000	0.927053000	3.258268000
H	-3.685399000	0.031764000	1.735499000
I	-1.389041000	0.089912000	-0.669761000

15. Acyl 13a

Rh	2.679313000	-0.926090000	-0.298912000
P	3.874916000	0.849528000	0.668709000
C	0.966688000	1.351996000	-3.575380000
C	1.353721000	0.138260000	-2.765487000
C	0.981573000	-1.127831000	-3.202066000
C	1.335015000	-2.362593000	-2.603750000
C	0.922333000	-3.626573000	-3.318711000
C	1.059491000	-0.786673000	0.826350000
C	4.066663000	1.068887000	2.489004000
C	3.435029000	0.196114000	3.384601000
C	3.536281000	0.404732000	4.762563000
C	4.271513000	1.484543000	5.256012000
C	4.909671000	2.356403000	4.367910000
C	4.804523000	2.153445000	2.992276000
C	3.408552000	2.551636000	0.109688000
C	4.378705000	3.497613000	-0.257181000
C	4.000953000	4.788816000	-0.632427000
C	2.651454000	5.148828000	-0.647887000
C	1.681436000	4.211636000	-0.282120000
C	2.054505000	2.920192000	0.092797000
C	5.580428000	0.652192000	0.007932000
C	6.678513000	0.330401000	0.815209000
C	7.928746000	0.091606000	0.237464000
C	8.090680000	0.166194000	-1.147715000
C	6.995375000	0.478228000	-1.960448000

C	5.746220000	0.714243000	-1.387318000
O	2.046906000	0.429138000	-1.702355000
N	2.001531000	-2.450924000	-1.469968000
O	0.684568000	0.270473000	1.296475000
H	2.870879000	-0.652647000	3.004917000
H	3.035885000	-0.276994000	5.448986000
H	4.345969000	1.649623000	6.330392000
H	5.485130000	3.200271000	4.745590000
H	5.293526000	2.847438000	2.310362000
H	1.296868000	2.191279000	0.368513000
H	0.626396000	4.484365000	-0.293982000
H	2.357248000	6.155512000	-0.944283000
H	4.766150000	5.511963000	-0.913478000
H	5.434654000	3.231326000	-0.257394000
H	6.560741000	0.261749000	1.895542000
H	8.776088000	-0.156642000	0.875059000
H	9.066169000	-0.021484000	-1.594993000
H	7.113314000	0.535673000	-3.041755000
H	4.893801000	0.953848000	-2.024176000
H	1.870447000	1.895325000	-3.881142000
H	0.392706000	1.078508000	-4.465601000
H	0.373982000	2.036612000	-2.954817000
H	1.261434000	-4.522555000	-2.788086000
H	-0.170756000	-3.664348000	-3.411628000
H	1.332927000	-3.640647000	-4.336076000
H	0.405722000	-1.180482000	-4.123892000
C	0.270803000	-2.069116000	1.021879000
H	0.927335000	-2.863670000	1.394604000
H	-0.552975000	-1.880421000	1.719402000
H	-0.123156000	-2.395589000	0.051531000
I	3.956100000	-2.812596000	1.176853000
H	2.207523000	-3.408375000	-1.172860000

16. Acyl 14a

Rh	2.689370000	-0.866021000	-0.314990000
P	3.883897000	0.826439000	0.678105000
C	0.904585000	1.249240000	-3.729645000
C	1.378852000	0.112121000	-2.854652000
C	1.116660000	-1.204281000	-3.292183000
C	1.469017000	-2.381206000	-2.632687000
C	1.136818000	-3.712687000	-3.262352000
C	1.057983000	-0.753486000	0.795927000

C	4.093777000	1.034943000	2.496687000
C	3.453795000	0.184486000	3.406718000
C	3.598989000	0.390208000	4.781129000
C	4.385965000	1.441606000	5.255163000
C	5.027506000	2.294300000	4.351031000
C	4.876868000	2.097770000	2.978451000
C	3.394785000	2.538480000	0.158349000
C	4.308082000	3.449444000	-0.394879000
C	3.895540000	4.735374000	-0.751473000
C	2.568550000	5.127264000	-0.562832000
C	1.653486000	4.228836000	-0.007462000
C	2.061004000	2.942790000	0.353961000
C	5.577584000	0.630589000	-0.010603000
C	6.691046000	0.337291000	0.786561000
C	7.933086000	0.092712000	0.195277000
C	8.073509000	0.135202000	-1.193409000
C	6.964050000	0.418077000	-1.996131000
C	5.721883000	0.656602000	-1.409558000
N	2.007576000	0.410490000	-1.729338000
O	2.076432000	-2.470593000	-1.491945000
O	0.788915000	0.189203000	1.514458000
H	2.849574000	-0.642249000	3.043400000
H	3.093855000	-0.275022000	5.479944000
H	4.497592000	1.600251000	6.327319000
H	5.641510000	3.117661000	4.712925000
H	5.371614000	2.775262000	2.283353000
H	1.345449000	2.247571000	0.790734000
H	0.617627000	4.527664000	0.147494000
H	2.249041000	6.129760000	-0.845392000
H	4.617445000	5.430581000	-1.177951000
H	5.345942000	3.160114000	-0.551052000
H	6.590807000	0.291025000	1.869580000
H	8.790962000	-0.136974000	0.825419000
H	9.043061000	-0.058474000	-1.651042000
H	7.063292000	0.446897000	-3.080273000
H	4.859831000	0.865048000	-2.043783000
H	2.074979000	1.416006000	-1.552233000
H	1.325594000	2.210349000	-3.413677000
H	1.177778000	1.070325000	-4.776052000
H	-0.190578000	1.320110000	-3.685966000
H	2.052899000	-4.307344000	-3.371970000
H	0.464805000	-4.274564000	-2.599736000
H	0.661480000	-3.596508000	-4.240866000
H	0.605371000	-1.306459000	-4.247418000

C	0.123776000	-1.939206000	0.640689000
H	0.672324000	-2.877994000	0.761534000
H	-0.682647000	-1.851770000	1.377200000
H	-0.295985000	-1.929372000	-0.373165000
I	3.894100000	-2.775137000	1.223291000

17. Acyl 16a

Rh	2.603846000	-0.898667000	-0.239125000
P	3.800982000	0.803144000	0.689095000
C	1.486556000	0.897985000	-4.216458000
C	1.670157000	-0.096071000	-3.092235000
C	1.235786000	-1.424009000	-3.335601000
C	1.300470000	-2.508570000	-2.464158000
C	0.765262000	-3.848542000	-2.913496000
C	3.403148000	-2.352894000	0.858910000
C	4.051982000	0.954789000	2.508516000
C	3.535806000	-0.013060000	3.378640000
C	3.746607000	0.089715000	4.755923000
C	4.473433000	1.163501000	5.272895000
C	4.985020000	2.138935000	4.410366000
C	4.773852000	2.038662000	3.035628000
C	3.272882000	2.503462000	0.188165000
C	4.203510000	3.500543000	-0.147060000
C	3.770865000	4.787367000	-0.474497000
C	2.407519000	5.092436000	-0.474071000
C	1.475827000	4.105619000	-0.142058000
C	1.903093000	2.818289000	0.187551000
C	5.503800000	0.662261000	-0.000921000
C	6.608851000	0.336155000	0.796880000
C	7.868921000	0.164779000	0.218385000
C	8.037393000	0.312412000	-1.160240000
C	6.937134000	0.625712000	-1.964403000
C	5.677524000	0.790703000	-1.390422000
N	2.210727000	0.296816000	-1.954177000
O	1.786001000	-2.520106000	-1.260726000
O	4.545189000	-2.547440000	0.454929000
H	2.946175000	-0.834212000	2.978658000
H	3.336190000	-0.667656000	5.422310000
H	4.636736000	1.246091000	6.346758000
H	5.547992000	2.981874000	4.808224000
H	5.174902000	2.806094000	2.375413000

H	1.169147000	2.051928000	0.439264000
H	0.410923000	4.334509000	-0.141463000
H	2.072110000	6.095954000	-0.734406000
H	4.503924000	5.550339000	-0.732895000
H	5.268815000	3.275307000	-0.159680000
H	6.491250000	0.215764000	1.872533000
H	8.719287000	-0.086881000	0.850480000
H	9.021078000	0.178220000	-1.608952000
H	7.057999000	0.735615000	-3.041270000
H	4.824603000	1.016331000	-2.029416000
H	2.419133000	1.298294000	-1.958644000
H	1.826983000	1.899846000	-3.931520000
H	2.045948000	0.576957000	-5.104834000
H	0.429519000	0.955341000	-4.506249000
H	1.553696000	-4.607578000	-2.827347000
H	-0.055450000	-4.156425000	-2.252163000
H	0.403355000	-3.820239000	-3.945642000
H	0.792176000	-1.614551000	-4.311660000
C	2.707524000	-3.323356000	1.777719000
H	3.423751000	-4.017397000	2.231595000
H	2.106740000	-2.808902000	2.534406000
H	2.000205000	-3.879421000	1.144521000
I	0.378038000	-0.618744000	1.204332000

[Rh(CH_3COCHCNPhCH_3)(CO)(PPh_3)] and products of the oxidative addition reaction [Rh(CH_3COCHCNPhCH_3)(CO)(PPh_3)] + CH_3I

1. [Rh(CH_3COCHCNPhCH_3)(CO)(PPh_3)] (O-trans-CO)

Rh	-1.152157000	0.668322000	1.900959000
N	-1.945294000	1.796209000	3.477775000
C	-1.368009000	2.866088000	4.020735000
C	-0.126608000	3.401481000	3.602368000
C	0.697657000	2.944952000	2.582020000
O	0.482363000	1.911308000	1.834604000
C	-2.595099000	-0.449878000	1.761452000
O	-3.527467000	-1.138689000	1.627273000
P	-0.082168000	-0.357243000	0.161981000
C	1.730416000	-0.601719000	0.433758000
C	2.226400000	-0.593781000	1.743979000
C	3.577184000	-0.846460000	1.987576000
C	4.443821000	-1.107825000	0.925298000

C	3.954191000	-1.123697000	-0.383686000
C	2.603389000	-0.877477000	-0.628750000
C	-0.196396000	0.708761000	-1.335323000
C	0.794619000	1.657420000	-1.626652000
C	0.617577000	2.564510000	-2.672990000
C	-0.550578000	2.540621000	-3.435976000
C	-1.549669000	1.608322000	-3.141830000
C	-1.379091000	0.704668000	-2.094659000
C	-0.599627000	-2.033325000	-0.407920000
C	-0.983285000	-2.968029000	0.565827000
C	-1.316834000	-4.271603000	0.200932000
C	-1.273603000	-4.655971000	-1.140719000
C	-0.886490000	-3.733316000	-2.114302000
C	-0.545604000	-2.429852000	-1.751128000
H	1.545729000	-0.378873000	2.567340000
H	3.952082000	-0.836109000	3.010778000
H	5.499218000	-1.303151000	1.115431000
H	4.624845000	-1.332745000	-1.216523000
H	2.230322000	-0.896894000	-1.651914000
H	1.700754000	1.695818000	-1.024303000
H	1.396225000	3.296389000	-2.886989000
H	-0.687564000	3.251042000	-4.251172000
H	-2.470693000	1.590710000	-3.723817000
H	-2.173817000	-0.003049000	-1.859310000
H	-1.025078000	-2.667479000	1.612692000
H	-1.619129000	-4.985233000	0.966458000
H	-1.543355000	-5.672444000	-1.427333000
H	-0.849646000	-4.026751000	-3.163053000
H	-0.246679000	-1.718114000	-2.519178000
H	0.215763000	4.281458000	4.141556000
C	1.978100000	3.682491000	2.266391000
H	2.158637000	4.512356000	2.956780000
H	2.824635000	2.984754000	2.304846000
H	1.928145000	4.074918000	1.241571000
C	-2.049709000	3.593314000	5.163122000
H	-3.002779000	4.030727000	4.837684000
H	-2.286426000	2.907076000	5.986368000
H	-1.408978000	4.394641000	5.541609000
C	-3.221893000	1.400120000	4.002646000
C	-3.302022000	0.431686000	5.009122000
C	-4.398809000	1.961270000	3.493870000
C	-4.544442000	0.041061000	5.510244000
C	-5.638855000	1.566936000	3.996764000
C	-5.717372000	0.608709000	5.009289000

H	-2.381805000	-0.014147000	5.386261000
H	-4.329448000	2.699257000	2.694778000
H	-4.594692000	-0.714012000	6.294270000
H	-6.548475000	2.008564000	3.590760000
H	-6.686684000	0.300429000	5.399158000

2. [Rh(CH_3COCHCNPhCH_3)(CO)(PPh_3)] (N-trans-CO)

Rh	-0.785263000	0.852018000	1.905610000
O	-1.789966000	2.183497000	3.172085000
C	-1.200385000	3.150897000	3.781639000
C	0.174651000	3.288751000	3.990240000
C	1.161298000	2.300769000	3.776464000
N	0.971199000	1.213540000	3.024073000
C	-2.419194000	0.288588000	1.270204000
O	-3.462877000	-0.140524000	0.978212000
P	0.168584000	-0.086162000	0.080904000
C	1.842530000	0.586142000	-0.334833000
C	2.079580000	1.936341000	-0.031773000
C	3.290571000	2.536371000	-0.375524000
C	4.281227000	1.794506000	-1.023239000
C	4.044726000	0.456182000	-1.343132000
C	2.829047000	-0.143545000	-1.010920000
C	-0.726058000	0.341081000	-1.488745000
C	-0.533601000	-0.393065000	-2.668402000
C	-1.162404000	-0.004133000	-3.850737000
C	-1.976033000	1.131651000	-3.874319000
C	-2.154154000	1.879785000	-2.710072000
C	-1.532543000	1.486955000	-1.524102000
C	0.295067000	-1.923007000	0.010572000
C	1.398698000	-2.600097000	0.551512000
C	1.403219000	-3.993635000	0.629086000
C	0.301902000	-4.729040000	0.188642000
C	-0.811815000	-4.061099000	-0.326994000
C	-0.817994000	-2.669889000	-0.413610000
H	1.312484000	2.511926000	0.486976000
H	3.460863000	3.585499000	-0.134343000
H	5.230803000	2.260432000	-1.286646000
H	4.807497000	-0.125530000	-1.859774000
H	2.655538000	-1.185270000	-1.274873000
H	0.099952000	-1.279166000	-2.663193000
H	-1.015087000	-0.590688000	-4.757205000
H	-2.468016000	1.432638000	-4.799252000

H	-2.782781000	2.769506000	-2.720097000
H	-1.673936000	2.063705000	-0.609344000
H	2.255211000	-2.041824000	0.928239000
H	2.270962000	-4.502707000	1.048420000
H	0.305653000	-5.817014000	0.256075000
H	-1.683518000	-4.623830000	-0.659576000
H	-1.697218000	-2.161786000	-0.808680000
H	0.488989000	4.164983000	4.552322000
C	2.468453000	2.510016000	4.516000000
H	2.399871000	3.386234000	5.166285000
H	2.718426000	1.634393000	5.129392000
H	3.302699000	2.656386000	3.818483000
C	-2.138506000	4.189438000	4.357358000
H	-2.799148000	4.566799000	3.566821000
H	-2.778324000	3.720006000	5.116623000
H	-1.600509000	5.026418000	4.813788000
C	1.891991000	0.120299000	3.166935000
C	1.451324000	-1.031583000	3.836979000
C	3.203860000	0.153774000	2.674009000
C	2.309908000	-2.111352000	4.035101000
C	4.058950000	-0.933300000	2.868759000
C	3.620577000	-2.067020000	3.554485000
H	0.425476000	-1.059966000	4.203310000
H	3.553571000	1.029748000	2.130996000
H	1.949592000	-2.991705000	4.566129000
H	5.075603000	-0.887682000	2.479258000
H	4.293291000	-2.909867000	3.710790000

3. Alkyl 1a

Rh	-0.991909000	0.854810000	1.832878000
O	-2.246491000	1.574689000	3.364112000
C	-1.835430000	2.405073000	4.265976000
C	-0.513778000	2.671338000	4.600430000
C	0.633382000	1.953707000	4.188566000
N	0.684247000	1.098460000	3.165454000
C	-2.610216000	0.631946000	0.938465000
O	-3.683874000	0.457162000	0.549156000
P	0.119435000	-0.061834000	-0.068623000
C	1.780079000	0.561108000	-0.598586000
C	2.138646000	1.893485000	-0.351276000
C	3.339148000	2.404755000	-0.847561000

C	4.194998000	1.591408000	-1.593027000
C	3.835844000	0.266510000	-1.855209000
C	2.631963000	-0.245917000	-1.370377000
C	-0.825544000	0.142883000	-1.655638000
C	-0.668140000	-0.789236000	-2.695792000
C	-1.311934000	-0.597703000	-3.918828000
C	-2.109740000	0.531170000	-4.126201000
C	-2.250889000	1.473197000	-3.105791000
C	-1.609895000	1.283554000	-1.879224000
C	0.293535000	-1.886095000	0.075720000
C	1.465152000	-2.472003000	0.575473000
C	1.535801000	-3.854296000	0.763622000
C	0.435627000	-4.662548000	0.472124000
C	-0.745692000	-4.081336000	0.000823000
C	-0.818984000	-2.702301000	-0.190630000
H	1.475993000	2.536035000	0.228989000
H	3.604588000	3.441855000	-0.645060000
H	5.134909000	1.988856000	-1.975274000
H	4.490515000	-0.373369000	-2.445015000
H	2.365297000	-1.275920000	-1.597322000
H	-0.048772000	-1.673178000	-2.556472000
H	-1.186533000	-1.334626000	-4.710872000
H	-2.612851000	0.677450000	-5.081468000
H	-2.858835000	2.363820000	-3.259545000
H	-1.706569000	2.044135000	-1.103324000
H	2.328506000	-1.854307000	0.819947000
H	2.456399000	-4.296357000	1.142901000
H	0.494344000	-5.740930000	0.616871000
H	-1.613155000	-4.701709000	-0.219191000
H	-1.746226000	-2.263962000	-0.557743000
H	-0.358491000	3.400206000	5.391748000
C	1.866789000	2.181658000	5.039415000
H	1.702925000	3.018547000	5.722710000
H	2.098479000	1.288660000	5.636518000
H	2.747253000	2.388843000	4.419574000
C	-2.948952000	3.096304000	5.013019000
H	-3.489455000	3.754577000	4.319241000
H	-3.666596000	2.353414000	5.383183000
H	-2.572062000	3.688888000	5.851509000
C	1.824136000	0.214694000	3.146573000
C	1.788613000	-0.984091000	3.876153000
C	2.999739000	0.558760000	2.471204000
C	2.894938000	-1.835110000	3.896228000
C	4.107495000	-0.292118000	2.495488000

C	4.057358000	-1.496917000	3.199647000
H	0.896346000	-1.236572000	4.445350000
H	3.052861000	1.507806000	1.944317000
H	2.847116000	-2.762428000	4.465827000
H	5.015438000	-0.002976000	1.967303000
H	4.921256000	-2.160497000	3.215710000
C	-1.307217000	-1.033183000	2.719454000
H	-1.543725000	-0.789756000	3.760376000
H	-2.142252000	-1.568648000	2.252469000
H	-0.388723000	-1.620812000	2.638067000
I	-0.886390000	3.674739000	1.000833000

4. Alkyl 2a

Rh	-1.089081000	0.647595000	2.014320000
N	-1.871910000	1.492995000	3.794509000
C	-1.137261000	2.089835000	4.721487000
C	0.267099000	2.252172000	4.652290000
C	1.130559000	1.842738000	3.641058000
O	0.811137000	1.222687000	2.552753000
C	-2.760774000	0.133128000	1.404003000
O	-3.807800000	-0.169605000	1.007442000
P	-0.045230000	-0.218422000	0.044983000
C	1.766875000	0.018111000	0.155081000
C	2.373610000	1.183003000	-0.334397000
C	3.741991000	1.391032000	-0.153730000
C	4.511179000	0.444507000	0.527374000
C	3.904584000	-0.705615000	1.040695000
C	2.537150000	-0.914897000	0.865250000
C	-0.532136000	0.571915000	-1.557081000
C	0.302536000	0.464726000	-2.683291000
C	-0.101461000	0.980668000	-3.914586000
C	-1.348878000	1.598615000	-4.043851000
C	-2.189860000	1.697735000	-2.934638000
C	-1.782652000	1.189822000	-1.698524000
C	-0.259892000	-2.009631000	-0.355577000
C	-1.419971000	-2.697098000	0.028908000
C	-1.618632000	-4.022213000	-0.363588000
C	-0.657222000	-4.678341000	-1.135128000
C	0.503480000	-4.001398000	-1.518507000
C	0.699370000	-2.673356000	-1.137217000
H	1.784266000	1.931530000	-0.861397000
H	4.204750000	2.295698000	-0.545615000

H	5.580237000	0.606342000	0.664656000
H	4.494908000	-1.440497000	1.586382000
H	2.065479000	-1.799509000	1.291113000
H	1.274260000	-0.018317000	-2.603770000
H	0.560367000	0.895150000	-4.775044000
H	-1.663464000	1.999491000	-5.006704000
H	-3.164976000	2.174492000	-3.025243000
H	-2.446904000	1.286098000	-0.843879000
H	-2.164936000	-2.216052000	0.655868000
H	-2.522856000	-4.543809000	-0.053379000
H	-0.809542000	-5.714300000	-1.434427000
H	1.261346000	-4.504699000	-2.116896000
H	1.609309000	-2.161743000	-1.444576000
H	0.725626000	2.760856000	5.496284000
C	2.607233000	2.124397000	3.773908000
H	2.840347000	2.646100000	4.706517000
H	3.163141000	1.178420000	3.734541000
H	2.945320000	2.729061000	2.921915000
C	-1.808442000	2.644647000	5.960354000
H	-2.529411000	3.430022000	5.698985000
H	-2.367126000	1.859198000	6.485219000
H	-1.063076000	3.063563000	6.640834000
C	-3.293428000	1.423568000	3.976319000
C	-3.868651000	0.299565000	4.580810000
C	-4.114091000	2.464569000	3.523253000
C	-5.254083000	0.222167000	4.733347000
C	-5.499482000	2.380160000	3.675783000
C	-6.073866000	1.259731000	4.282356000
H	-3.220362000	-0.510525000	4.913735000
H	-3.660936000	3.337536000	3.054419000
H	-5.692875000	-0.655562000	5.205943000
H	-6.129708000	3.194804000	3.321656000
H	-7.154769000	1.195398000	4.402076000
C	-1.221918000	2.600788000	1.214477000
H	-0.539495000	2.703450000	0.364848000
H	-2.244089000	2.854409000	0.913343000
H	-0.900881000	3.242054000	2.041870000
I	-0.661133000	-1.801961000	3.488237000

5. Alkyl 3a

Rh	-1.109200000	-0.505746000	2.005951000
N	-2.015017000	-1.288346000	3.815444000

C	-1.296155000	-1.654279000	4.864361000
C	0.121683000	-1.597078000	4.943790000
C	1.040042000	-1.213952000	3.975337000
O	0.794113000	-0.802962000	2.772514000
C	-1.274416000	1.281893000	2.811861000
O	-1.498856000	2.254437000	3.390989000
P	-0.011471000	0.188902000	0.034636000
C	1.786698000	-0.261351000	-0.027799000
C	2.178947000	-1.560050000	0.333883000
C	3.508702000	-1.960962000	0.201382000
C	4.467671000	-1.070153000	-0.289328000
C	4.085133000	0.222660000	-0.653423000
C	2.752969000	0.624243000	-0.526685000
C	-0.550788000	-0.391957000	-1.639541000
C	0.082669000	0.149401000	-2.770541000
C	-0.241161000	-0.306016000	-4.048528000
C	-1.190036000	-1.320039000	-4.211982000
C	-1.816546000	-1.868575000	-3.091733000
C	-1.502620000	-1.405486000	-1.811078000
C	-0.010974000	2.024279000	-0.084966000
C	0.781863000	2.757761000	0.813750000
C	0.735507000	4.150904000	0.819764000
C	-0.114578000	4.827127000	-0.060344000
C	-0.919382000	4.102837000	-0.942827000
C	-0.870618000	2.706603000	-0.955968000
H	1.447950000	-2.258381000	0.731635000
H	3.796539000	-2.971872000	0.488420000
H	5.506957000	-1.382690000	-0.388084000
H	4.821640000	0.925395000	-1.041129000
H	2.475860000	1.634976000	-0.819958000
H	0.831925000	0.931480000	-2.659340000
H	0.253101000	0.129569000	-4.915668000
H	-1.438070000	-1.680741000	-5.209867000
H	-2.557853000	-2.657840000	-3.209200000
H	-2.012257000	-1.823969000	-0.948440000
H	1.437357000	2.237837000	1.512239000
H	1.360923000	4.708156000	1.515628000
H	-0.153539000	5.915939000	-0.053486000
H	-1.592587000	4.622335000	-1.623380000
H	-1.508216000	2.150241000	-1.640783000
H	0.547183000	-1.918678000	5.890913000
C	2.516134000	-1.256303000	4.299220000
H	2.698469000	-1.654524000	5.301497000
H	2.935680000	-0.243835000	4.231727000

H	3.040590000	-1.873086000	3.558199000
C	-1.988823000	-2.182186000	6.105738000
H	-2.544027000	-3.101981000	5.880722000
H	-2.719407000	-1.455997000	6.483964000
H	-1.258892000	-2.394663000	6.890749000
C	-3.439366000	-1.419246000	3.904025000
C	-4.214752000	-0.352754000	4.374327000
C	-4.065968000	-2.614103000	3.532012000
C	-5.599408000	-0.484446000	4.482078000
C	-5.452479000	-2.740876000	3.635029000
C	-6.224356000	-1.678530000	4.111859000
H	-3.726579000	0.579484000	4.656541000
H	-3.460973000	-3.443236000	3.168321000
H	-6.191554000	0.351238000	4.853142000
H	-5.929474000	-3.676268000	3.344547000
H	-7.305853000	-1.779864000	4.194084000
C	-1.114917000	-2.528998000	1.388125000
H	-0.644555000	-3.082483000	2.212242000
H	-0.574520000	-2.742041000	0.458816000
H	-2.157978000	-2.850046000	1.272785000
I	-3.502591000	0.055173000	0.756065000

6. Alkyl 4a

Rh	-0.858607000	0.713205000	1.920979000
O	-1.941053000	1.602243000	3.520640000
C	-1.479153000	2.616946000	4.167800000
C	-0.139868000	3.001251000	4.248602000
C	0.967935000	2.209819000	3.868179000
N	0.924682000	1.181446000	3.020365000
C	-1.066862000	-1.064763000	2.744905000
O	-1.335989000	-2.076760000	3.227498000
P	0.129309000	-0.034563000	-0.055983000
C	1.819006000	0.622517000	-0.444709000
C	2.115985000	1.964818000	-0.156687000
C	3.347199000	2.513076000	-0.520892000
C	4.303183000	1.726380000	-1.168150000
C	4.014036000	0.391468000	-1.460857000
C	2.778633000	-0.154909000	-1.110821000
C	-0.703489000	0.295227000	-1.688870000
C	-0.239655000	-0.407271000	-2.814893000
C	-0.774662000	-0.157394000	-4.078783000
C	-1.771705000	0.809135000	-4.240564000

C	-2.231562000	1.516459000	-3.129334000
C	-1.704382000	1.259263000	-1.860884000
C	0.295605000	-1.867663000	-0.054697000
C	1.383899000	-2.496815000	0.569225000
C	1.430654000	-3.888656000	0.665325000
C	0.385300000	-4.665505000	0.159968000
C	-0.713027000	-4.043228000	-0.439664000
C	-0.760306000	-2.652375000	-0.546084000
H	1.395457000	2.580268000	0.372948000
H	3.561261000	3.555045000	-0.285707000
H	5.267534000	2.151839000	-1.444503000
H	4.750181000	-0.230828000	-1.967784000
H	2.573969000	-1.195412000	-1.352370000
H	0.536760000	-1.162828000	-2.711258000
H	-0.408273000	-0.719482000	-4.936470000
H	-2.187932000	1.007343000	-5.227794000
H	-3.011452000	2.268635000	-3.240100000
H	-2.095706000	1.800098000	-1.006282000
H	2.199258000	-1.905047000	0.984036000
H	2.286763000	-4.364337000	1.142347000
H	0.423373000	-5.751717000	0.238397000
H	-1.538198000	-4.639154000	-0.826833000
H	-1.625470000	-2.176613000	-1.005835000
H	0.078742000	3.880492000	4.849351000
C	2.276587000	2.554341000	4.553233000
H	2.186064000	3.510505000	5.075104000
H	2.537736000	1.784961000	5.293627000
H	3.104756000	2.608208000	3.837403000
C	-2.520977000	3.395024000	4.935801000
H	-3.278129000	3.777642000	4.238941000
H	-3.036489000	2.724973000	5.636500000
H	-2.083006000	4.228489000	5.492890000
C	1.960184000	0.183603000	3.162398000
C	1.749426000	-0.875794000	4.058879000
C	3.181794000	0.259039000	2.483888000
C	2.723007000	-1.855592000	4.247787000
C	4.159253000	-0.720769000	2.681431000
C	3.933189000	-1.786324000	3.552766000
H	0.818465000	-0.920197000	4.622270000
H	3.379534000	1.089210000	1.810983000
H	2.536058000	-2.669747000	4.946637000
H	5.105857000	-0.642704000	2.147641000
H	4.695865000	-2.550258000	3.698166000
C	-0.913070000	2.670808000	1.099984000

H	-0.656995000	2.769962000	0.040874000
H	-1.945247000	3.015246000	1.245435000
H	-0.229187000	3.292648000	1.691846000
I	-3.418231000	0.166003000	0.993347000

7. Alkyl 5a

Rh	-0.875429000	0.764196000	2.132604000
O	-1.788936000	1.578561000	3.816664000
C	-1.163708000	1.874548000	4.913218000
C	0.207665000	1.849173000	5.127314000
C	1.247843000	1.507363000	4.219248000
N	1.057823000	1.108719000	2.969362000
C	-2.530013000	0.241391000	1.457571000
O	-3.560820000	-0.137952000	1.090897000
P	-1.027191000	3.063091000	0.969829000
C	0.238575000	4.343567000	1.409638000
C	0.525040000	4.530424000	2.771189000
C	1.448481000	5.494501000	3.176601000
C	2.103570000	6.283224000	2.226585000
C	1.819590000	6.108094000	0.870547000
C	0.890420000	5.147389000	0.462963000
C	-2.591492000	3.987442000	1.331732000
C	-2.747897000	5.307456000	0.873491000
C	-3.921161000	6.013819000	1.133969000
C	-4.954525000	5.413896000	1.861773000
C	-4.804731000	4.107347000	2.327719000
C	-3.628435000	3.397247000	2.065891000
C	-0.977024000	3.023568000	-0.869099000
C	0.220585000	2.657667000	-1.506667000
C	0.286053000	2.576318000	-2.896935000
C	-0.851216000	2.825601000	-3.670140000
C	-2.054361000	3.156109000	-3.043103000
C	-2.117428000	3.257173000	-1.650579000
H	0.030916000	3.916919000	3.524318000
H	1.660481000	5.625415000	4.237413000
H	2.830105000	7.031371000	2.542371000
H	2.317384000	6.723507000	0.122112000
H	0.677820000	5.028564000	-0.597814000
H	-1.949028000	5.786988000	0.308647000
H	-4.027149000	7.035210000	0.770954000
H	-5.869549000	5.968192000	2.069664000
H	-5.600003000	3.636775000	2.904442000

H	-3.510942000	2.395985000	2.469287000
H	1.107254000	2.431728000	-0.916751000
H	1.225081000	2.303796000	-3.376377000
H	-0.800685000	2.754835000	-4.756254000
H	-2.949863000	3.341823000	-3.634898000
H	-3.060445000	3.525555000	-1.176823000
H	0.535332000	2.152181000	6.118368000
C	2.653258000	1.644897000	4.765356000
H	2.634329000	2.078826000	5.768180000
H	3.149888000	0.667318000	4.815337000
H	3.265038000	2.278806000	4.110842000
C	-2.085239000	2.298533000	6.031881000
H	-2.707745000	3.139942000	5.700688000
H	-2.763246000	1.472068000	6.282488000
H	-1.529104000	2.590650000	6.927218000
C	2.219361000	0.762461000	2.195286000
C	2.791698000	-0.510290000	2.311111000
C	2.783126000	1.690977000	1.315153000
C	3.914738000	-0.846947000	1.553615000
C	3.903874000	1.350440000	0.555406000
C	4.474188000	0.080370000	0.670523000
H	2.351534000	-1.235190000	2.994483000
H	2.344808000	2.685497000	1.240342000
H	4.348995000	-1.840990000	1.651284000
H	4.334559000	2.084040000	-0.125508000
H	5.348440000	-0.185009000	0.077256000
C	-0.786603000	-1.042911000	3.233478000
H	-0.562962000	-0.731063000	4.260063000
H	-1.738933000	-1.584206000	3.198719000
H	0.013977000	-1.667964000	2.826935000
I	-0.044271000	-0.648591000	-0.071052000

8. Alkyl 6a

Rh	-0.949504000	0.809028000	2.014645000
N	-2.402511000	1.395119000	3.400253000
C	-2.149164000	2.154755000	4.457227000
C	-0.857323000	2.589137000	4.849315000
C	0.366726000	2.335769000	4.234800000
O	0.560095000	1.634210000	3.164772000
C	-2.144817000	0.196527000	0.738203000
O	-2.862961000	-0.116851000	-0.113706000
P	-0.506244000	-1.486043000	3.073342000

C	1.235768000	-1.906323000	3.536910000
C	2.275655000	-0.989102000	3.335538000
C	3.578622000	-1.305582000	3.734133000
C	3.853142000	-2.536029000	4.332507000
C	2.818694000	-3.456021000	4.536172000
C	1.517440000	-3.141630000	4.146173000
C	-1.024181000	-2.853258000	1.955871000
C	-0.095035000	-3.665745000	1.292055000
C	-0.527069000	-4.634608000	0.381985000
C	-1.889059000	-4.802493000	0.125097000
C	-2.821764000	-3.988217000	0.775216000
C	-2.392212000	-3.014771000	1.676187000
C	-1.370812000	-1.869616000	4.667110000
C	-1.278666000	-0.912905000	5.692278000
C	-1.886634000	-1.135194000	6.927872000
C	-2.600301000	-2.315460000	7.157090000
C	-2.690388000	-3.274617000	6.146445000
C	-2.076390000	-3.056904000	4.909832000
H	2.066693000	-0.026065000	2.878470000
H	4.379027000	-0.583590000	3.575405000
H	4.868841000	-2.779360000	4.643853000
H	3.023690000	-4.417775000	5.004681000
H	0.718501000	-3.862353000	4.318992000
H	0.970003000	-3.542403000	1.480253000
H	0.207867000	-5.256854000	-0.126812000
H	-2.224278000	-5.559961000	-0.582491000
H	-3.886039000	-4.106634000	0.576297000
H	-3.130358000	-2.377890000	2.164285000
H	-0.733888000	0.016967000	5.525903000
H	-1.806615000	-0.382605000	7.711823000
H	-3.081152000	-2.486396000	8.119713000
H	-3.235693000	-4.201819000	6.317787000
H	-2.153366000	-3.818559000	4.136144000
H	-0.822239000	3.198422000	5.748618000
C	1.630221000	2.900028000	4.836417000
H	1.432518000	3.469649000	5.748742000
H	2.326968000	2.082242000	5.063984000
H	2.124790000	3.550871000	4.103544000
C	-3.295928000	2.593304000	5.342441000
H	-4.018415000	3.196591000	4.777693000
H	-3.840721000	1.723718000	5.732421000
H	-2.924688000	3.184482000	6.183114000
C	-3.766330000	1.072979000	3.074513000
C	-4.343984000	-0.111552000	3.546270000

C	-4.513853000	1.927939000	2.253584000
C	-5.654603000	-0.440193000	3.193134000
C	-5.823437000	1.594612000	1.904556000
C	-6.398215000	0.409171000	2.370420000
H	-3.760758000	-0.764965000	4.194155000
H	-4.062750000	2.848044000	1.884411000
H	-6.094885000	-1.364455000	3.565902000
H	-6.394236000	2.264830000	1.263314000
H	-7.419207000	0.149386000	2.093828000
C	-1.109674000	2.752284000	1.186584000
H	-0.077327000	3.108792000	1.117044000
H	-1.580639000	2.759849000	0.197480000
H	-1.685441000	3.360108000	1.893135000
I	0.931430000	0.270332000	0.055895000

9. Alkyl 7a

Rh	-1.266996000	0.495325000	1.860289000
N	-1.600784000	1.267808000	3.904676000
C	-0.716683000	2.015412000	4.542020000
C	0.538235000	2.410710000	3.999487000
C	1.162156000	1.984701000	2.832627000
O	0.667165000	1.227134000	1.901624000
C	-2.886441000	-0.392999000	1.750288000
O	-3.868836000	-1.003829000	1.682358000
P	-1.978601000	2.440360000	0.714386000
C	-3.009483000	2.188756000	-0.802472000
C	-3.538450000	0.944298000	-1.167155000
C	-4.333944000	0.818524000	-2.309139000
C	-4.606727000	1.934549000	-3.100220000
C	-4.079911000	3.180537000	-2.746491000
C	-3.285810000	3.307530000	-1.608162000
C	-0.606031000	3.501798000	0.082621000
C	-0.666137000	4.902068000	0.152025000
C	0.350378000	5.681421000	-0.405209000
C	1.434564000	5.073011000	-1.039974000
C	1.496762000	3.679001000	-1.119505000
C	0.486117000	2.895864000	-0.561588000
C	-3.008711000	3.571929000	1.732488000
C	-4.393895000	3.670756000	1.533053000
C	-5.169827000	4.478456000	2.368166000
C	-4.571371000	5.194057000	3.406608000
C	-3.191915000	5.096175000	3.612635000

C	-2.414835000	4.284692000	2.787700000
H	-3.321865000	0.058115000	-0.579546000
H	-4.732549000	-0.157714000	-2.579602000
H	-5.222566000	1.834969000	-3.993174000
H	-4.283479000	4.057190000	-3.359408000
H	-2.880524000	4.284264000	-1.347300000
H	-1.506120000	5.394401000	0.637976000
H	0.287985000	6.766863000	-0.342151000
H	2.228335000	5.682311000	-1.471451000
H	2.339243000	3.195484000	-1.612295000
H	0.557240000	1.814477000	-0.618708000
H	-4.872855000	3.120248000	0.725126000
H	-6.243557000	4.546402000	2.201244000
H	-5.176921000	5.825907000	4.055627000
H	-2.719002000	5.651597000	4.421681000
H	-1.341867000	4.206812000	2.962848000
H	1.131256000	3.065436000	4.634293000
C	2.591059000	2.398618000	2.573619000
H	2.972112000	3.063309000	3.354346000
H	3.224664000	1.502732000	2.525810000
H	2.660070000	2.898565000	1.598424000
C	-0.996054000	2.524931000	5.941811000
H	-2.062169000	2.728562000	6.091090000
H	-0.692865000	1.784561000	6.694205000
H	-0.421392000	3.438054000	6.125734000
C	-2.725630000	0.765989000	4.636694000
C	-2.543881000	-0.207141000	5.632064000
C	-4.023572000	1.204070000	4.345355000
C	-3.640000000	-0.718967000	6.328357000
C	-5.117711000	0.689964000	5.044115000
C	-4.932945000	-0.272457000	6.040040000
H	-1.538775000	-0.568481000	5.843034000
H	-4.171305000	1.960070000	3.575937000
H	-3.481064000	-1.475163000	7.096545000
H	-6.119687000	1.048147000	4.808478000
H	-5.787714000	-0.673533000	6.583671000
C	-0.656738000	-0.353106000	0.005227000
H	-1.030081000	-1.379210000	-0.087918000
H	-0.969322000	0.224143000	-0.871725000
H	0.435845000	-0.364255000	0.088578000
I	-0.285394000	-1.857459000	3.001188000

10. Alkyl 8a

Rh	-1.012118000	0.709441000	2.004271000
O	-1.936382000	1.624397000	3.664146000
C	-1.347724000	1.891771000	4.787150000
C	0.010638000	1.853938000	5.062500000
C	1.105114000	1.619688000	4.185306000
N	1.005830000	1.173957000	2.940350000
C	-1.030051000	2.378979000	1.196763000
O	-1.085250000	3.471507000	0.821746000
P	-0.488038000	-0.425149000	-0.021150000
C	-0.241012000	0.814456000	-1.365143000
C	-1.353027000	1.511605000	-1.869412000
C	-1.188274000	2.512055000	-2.825664000
C	0.091463000	2.839198000	-3.285833000
C	1.201726000	2.161935000	-2.779185000
C	1.038816000	1.155612000	-1.823231000
C	-1.820991000	-1.525703000	-0.706900000
C	-2.116008000	-1.546939000	-2.078373000
C	-3.047587000	-2.455386000	-2.588432000
C	-3.688909000	-3.358652000	-1.739255000
C	-3.386531000	-3.354312000	-0.374739000
C	-2.458997000	-2.447076000	0.137801000
C	0.953525000	-1.575103000	-0.157309000
C	1.530873000	-2.149698000	0.979849000
C	2.530858000	-3.117796000	0.849870000
C	2.965755000	-3.513399000	-0.414724000
C	2.390999000	-2.943414000	-1.556150000
C	1.385182000	-1.986861000	-1.430725000
H	-2.356743000	1.273097000	-1.520592000
H	-2.061272000	3.036686000	-3.210701000
H	0.220314000	3.621034000	-4.033527000
H	2.203303000	2.414362000	-3.125701000
H	1.914288000	0.641354000	-1.431954000
H	-1.624098000	-0.858538000	-2.762265000
H	-3.267103000	-2.452365000	-3.655183000
H	-4.417160000	-4.064120000	-2.137848000
H	-3.874296000	-4.058692000	0.298152000
H	-2.229172000	-2.458799000	1.203512000
H	1.182878000	-1.859095000	1.968868000
H	2.970053000	-3.558943000	1.744159000
H	3.746410000	-4.267268000	-0.515622000
H	2.718537000	-3.250244000	-2.548619000
H	0.934711000	-1.568780000	-2.329068000

H	0.285786000	2.156202000	6.069979000
C	2.460925000	1.957107000	4.782518000
H	2.336560000	2.514537000	5.714435000
H	3.030270000	1.044701000	5.001370000
H	3.065659000	2.552458000	4.087446000
C	-2.309531000	2.312531000	5.874118000
H	-2.915326000	3.159317000	5.526473000
H	-2.998716000	1.485012000	6.088192000
H	-1.787855000	2.594356000	6.793281000
C	2.214352000	1.118763000	2.173898000
C	2.458357000	2.072994000	1.177773000
C	3.183756000	0.136524000	2.420845000
C	3.652960000	2.055013000	0.454901000
C	4.376588000	0.119608000	1.697579000
C	4.619348000	1.080007000	0.712070000
H	1.716666000	2.846795000	0.984566000
H	2.998844000	-0.610469000	3.191532000
H	3.827213000	2.810480000	-0.310794000
H	5.119212000	-0.650151000	1.905281000
H	5.551484000	1.066664000	0.148541000
C	-3.048964000	0.491649000	1.455804000
H	-3.379645000	-0.497614000	1.784482000
H	-3.557528000	1.273636000	2.030860000
H	-3.245610000	0.613693000	0.384828000
I	-1.119998000	-1.592824000	3.575640000

11. Alkyl 9a

Rh	-1.338439000	0.760882000	1.690421000
N	-2.176439000	1.578312000	3.494907000
C	-1.463883000	1.804245000	4.596092000
C	-0.086926000	1.532760000	4.762420000
C	0.808237000	0.921755000	3.878706000
O	0.554803000	0.496795000	2.694675000
C	-1.913243000	-0.906626000	2.250843000
O	-2.268740000	-1.908254000	2.711490000
P	-0.278209000	-0.058954000	-0.265908000
C	1.507730000	-0.312926000	0.060192000
C	2.416938000	0.724799000	-0.188475000
C	3.760790000	0.578492000	0.157486000
C	4.205897000	-0.599155000	0.763343000
C	3.298423000	-1.626686000	1.034344000
C	1.953996000	-1.480715000	0.695175000

C	-0.318860000	0.912180000	-1.842095000
C	0.570306000	0.565595000	-2.873648000
C	0.522350000	1.228634000	-4.100004000
C	-0.419715000	2.238609000	-4.315334000
C	-1.308445000	2.584912000	-3.295985000
C	-1.258100000	1.928208000	-2.063998000
C	-0.884173000	-1.715801000	-0.836283000
C	-2.224305000	-2.083525000	-0.640787000
C	-2.707572000	-3.299885000	-1.128693000
C	-1.857612000	-4.164989000	-1.820071000
C	-0.523452000	-3.804227000	-2.027702000
C	-0.040385000	-2.587520000	-1.545491000
H	2.075052000	1.652258000	-0.645012000
H	4.459030000	1.389622000	-0.045645000
H	5.256376000	-0.713402000	1.029772000
H	3.634726000	-2.542863000	1.517961000
H	1.254295000	-2.282426000	0.926551000
H	1.312366000	-0.215744000	-2.722825000
H	1.223905000	0.953638000	-4.886146000
H	-0.458019000	2.755286000	-5.273885000
H	-2.042055000	3.374690000	-3.452050000
H	-1.938082000	2.225928000	-1.272475000
H	-2.903848000	-1.419148000	-0.112122000
H	-3.750165000	-3.568502000	-0.964633000
H	-2.232506000	-5.116140000	-2.196488000
H	0.147890000	-4.471368000	-2.566297000
H	1.002484000	-2.327734000	-1.713741000
H	0.325393000	1.815506000	5.727917000
C	2.240011000	0.720051000	4.324056000
H	2.400850000	1.040654000	5.357691000
H	2.512772000	-0.337759000	4.221101000
H	2.905453000	1.287723000	3.659880000
C	-2.152296000	2.415449000	5.801703000
H	-2.631605000	3.366979000	5.538493000
H	-2.942878000	1.753447000	6.178623000
H	-1.430753000	2.591392000	6.603319000
C	-3.580353000	1.853762000	3.523953000
C	-4.483260000	0.874372000	3.956486000
C	-4.064239000	3.099818000	3.107415000
C	-5.852770000	1.143373000	3.987723000
C	-5.434199000	3.366881000	3.141643000
C	-6.333367000	2.391915000	3.582526000
H	-4.106098000	-0.096951000	4.276528000
H	-3.355541000	3.850575000	2.759021000

H	-6.543725000	0.375745000	4.333661000
H	-5.799122000	4.342262000	2.822123000
H	-7.401823000	2.602807000	3.609461000
C	-3.152996000	1.069112000	0.624530000
H	-3.129531000	0.663237000	-0.392556000
H	-3.984117000	0.608423000	1.173566000
H	-3.304026000	2.153854000	0.582944000
I	-0.478775000	3.348531000	1.133999000

12. Alkyl 10a

Rh	-1.119280000	0.772111000	1.871124000
O	-1.764626000	1.413717000	3.834585000
C	-0.968492000	1.801166000	4.770519000
C	0.422593000	1.853997000	4.705992000
C	1.292494000	1.551017000	3.632062000
N	0.928746000	1.098857000	2.428603000
C	-2.842544000	0.654781000	1.190506000
O	-3.875541000	0.598441000	0.670401000
P	-1.202060000	-1.435952000	2.707412000
C	-0.817247000	-2.773986000	1.504299000
C	-1.526758000	-2.781045000	0.290850000
C	-1.305233000	-3.783366000	-0.650910000
C	-0.365685000	-4.787803000	-0.397850000
C	0.339874000	-4.789635000	0.806085000
C	0.113990000	-3.790621000	1.756907000
C	-2.898786000	-1.882134000	3.286806000
C	-3.333185000	-3.216112000	3.227472000
C	-4.599900000	-3.564046000	3.698617000
C	-5.442694000	-2.588102000	4.236407000
C	-5.010673000	-1.262049000	4.307979000
C	-3.745112000	-0.906261000	3.836950000
C	-0.146011000	-1.768669000	4.180297000
C	1.254951000	-1.773829000	4.075034000
C	2.049368000	-2.032549000	5.190764000
C	1.457558000	-2.272383000	6.433728000
C	0.066867000	-2.255557000	6.551489000
C	-0.731965000	-2.006286000	5.433598000
H	-2.258583000	-2.003576000	0.078997000
H	-1.863077000	-3.776110000	-1.585790000
H	-0.187366000	-5.567190000	-1.137689000
H	1.065496000	-5.573999000	1.015897000
H	0.657202000	-3.817793000	2.699168000

H	-2.686311000	-3.987073000	2.811368000
H	-4.927107000	-4.600991000	3.641199000
H	-6.433320000	-2.861319000	4.598273000
H	-5.660409000	-0.496666000	4.730320000
H	-3.402434000	0.124830000	3.915361000
H	1.736763000	-1.576664000	3.122009000
H	3.133657000	-2.042550000	5.087847000
H	2.078882000	-2.471395000	7.306460000
H	-0.404926000	-2.440445000	7.515576000
H	-1.815097000	-2.004915000	5.541727000
H	0.919139000	2.176688000	5.618502000
C	2.761496000	1.774149000	3.941373000
H	2.919237000	1.750790000	5.023317000
H	3.396660000	1.017688000	3.466542000
H	3.094275000	2.754180000	3.574130000
C	-1.663149000	2.230088000	6.042402000
H	-2.358446000	3.050138000	5.819823000
H	-2.261228000	1.396117000	6.433795000
H	-0.957127000	2.556256000	6.811538000
C	1.940134000	1.062506000	1.411814000
C	2.426420000	-0.147739000	0.901971000
C	2.448682000	2.263367000	0.892942000
C	3.427419000	-0.161326000	-0.071026000
C	3.446249000	2.250451000	-0.082382000
C	3.947504000	1.037734000	-0.564009000
H	2.015453000	-1.090560000	1.257235000
H	2.045489000	3.206482000	1.258607000
H	3.797870000	-1.114430000	-0.446582000
H	3.831182000	3.194032000	-0.467440000
H	4.729207000	1.026914000	-1.322433000
C	-0.532854000	0.282821000	-0.112027000
H	0.105271000	1.109351000	-0.442725000
H	0.026056000	-0.658834000	-0.105901000
H	-1.388235000	0.195532000	-0.795181000
I	-1.292310000	3.454021000	1.064568000

13. Alkyl 11a

Rh	-1.046777000	0.757369000	1.723642000
N	-1.497502000	1.887386000	3.461341000
C	-0.647245000	2.766082000	3.987987000
C	0.705569000	2.946578000	3.607635000
C	1.466152000	2.251980000	2.661441000

O	1.050234000	1.311114000	1.890680000
C	-1.497328000	2.221732000	0.655132000
O	-1.820797000	3.106901000	-0.013916000
P	-0.575403000	-1.380647000	2.920588000
C	-0.379403000	-1.291705000	4.761662000
C	0.340125000	-0.207498000	5.288729000
C	0.582764000	-0.116546000	6.660688000
C	0.109816000	-1.107408000	7.524637000
C	-0.591233000	-2.198940000	7.005684000
C	-0.829109000	-2.295991000	5.632916000
C	0.983081000	-2.277309000	2.473054000
C	1.158989000	-3.608222000	2.886863000
C	2.358202000	-4.273510000	2.630399000
C	3.399108000	-3.613363000	1.969582000
C	3.232695000	-2.287226000	1.567049000
C	2.029747000	-1.618296000	1.813449000
C	-1.895872000	-2.628164000	2.650040000
C	-3.072865000	-2.604363000	3.415825000
C	-4.115882000	-3.488922000	3.139574000
C	-4.005462000	-4.395437000	2.082418000
C	-2.849220000	-4.407098000	1.298588000
C	-1.801583000	-3.527777000	1.577343000
H	0.715689000	0.573990000	4.629837000
H	1.140862000	0.732819000	7.053654000
H	0.292985000	-1.033310000	8.596227000
H	-0.951706000	-2.984091000	7.668764000
H	-1.366822000	-3.160133000	5.248651000
H	0.360147000	-4.133012000	3.408640000
H	2.479082000	-5.307077000	2.951557000
H	4.336776000	-4.132599000	1.772916000
H	4.040827000	-1.766403000	1.054330000
H	1.907892000	-0.583196000	1.503506000
H	-3.181501000	-1.894013000	4.233471000
H	-5.018401000	-3.464084000	3.748764000
H	-4.820478000	-5.085408000	1.866769000
H	-2.758065000	-5.101537000	0.464782000
H	-0.908951000	-3.539910000	0.954452000
H	1.239406000	3.710223000	4.167851000
C	2.931630000	2.591322000	2.511057000
H	3.240155000	3.409209000	3.168558000
H	3.536925000	1.702759000	2.735785000
H	3.137370000	2.865646000	1.468507000
C	-1.121618000	3.649014000	5.125290000
H	-1.975934000	4.264042000	4.814679000

H	-1.459330000	3.041322000	5.974909000
H	-0.315907000	4.306592000	5.460279000
C	-2.850287000	1.876440000	3.940515000
C	-3.225182000	1.019705000	4.981869000
C	-3.812061000	2.711005000	3.358014000
C	-4.545328000	1.000250000	5.435797000
C	-5.129816000	2.691937000	3.815424000
C	-5.502863000	1.835152000	4.855005000
H	-2.471816000	0.377846000	5.437242000
H	-3.523519000	3.376357000	2.544696000
H	-4.824049000	0.331486000	6.249392000
H	-5.867251000	3.348033000	3.355199000
H	-6.532759000	1.818622000	5.209171000
C	-3.051445000	0.116525000	1.460104000
H	-3.659229000	0.889514000	0.971173000
H	-3.041860000	-0.787065000	0.840039000
H	-3.470767000	-0.098355000	2.449289000
I	-0.440833000	-0.468870000	-0.667109000

14. Alkyl 12a

Rh	-1.005436000	-0.708711000	1.958230000
N	-2.294682000	-1.591016000	3.561022000
C	-1.827292000	-2.501324000	4.394840000
C	-0.452542000	-2.875533000	4.492758000
C	0.659470000	-2.360645000	3.841855000
O	0.692214000	-1.432113000	2.929325000
C	-1.181885000	-2.274349000	0.945797000
O	-1.255981000	-3.235163000	0.308125000
P	-0.684782000	1.488770000	3.074914000
C	-1.628032000	2.847258000	2.276455000
C	-2.996279000	3.001336000	2.550643000
C	-3.742903000	3.980166000	1.896392000
C	-3.142649000	4.795247000	0.933613000
C	-1.791389000	4.622123000	0.626149000
C	-1.037216000	3.654876000	1.293984000
C	-1.130531000	1.602560000	4.868868000
C	-1.686821000	2.754904000	5.445061000
C	-1.938839000	2.806083000	6.817425000
C	-1.627642000	1.714906000	7.632394000
C	-1.055131000	0.572108000	7.068368000
C	-0.808409000	0.515266000	5.695664000
C	1.054126000	2.135630000	3.130544000

C	2.150337000	1.284416000	2.931632000
C	3.454093000	1.775409000	3.051693000
C	3.676341000	3.115371000	3.371850000
C	2.587096000	3.966565000	3.583565000
C	1.285078000	3.479995000	3.469950000
H	-3.486545000	2.351554000	3.273137000
H	-4.800627000	4.094813000	2.129556000
H	-3.728284000	5.555940000	0.418486000
H	-1.316448000	5.242117000	-0.132875000
H	0.017193000	3.539442000	1.049054000
H	-1.930134000	3.617130000	4.827730000
H	-2.376239000	3.705387000	7.248798000
H	-1.826278000	1.756875000	8.702937000
H	-0.802422000	-0.281295000	7.696962000
H	-0.367571000	-0.384918000	5.270759000
H	1.984982000	0.235635000	2.701760000
H	4.297131000	1.103516000	2.893826000
H	4.693246000	3.496210000	3.462166000
H	2.749515000	5.012091000	3.841995000
H	0.448241000	4.154452000	3.643636000
H	-0.237879000	-3.645715000	5.229510000
C	2.033209000	-2.876386000	4.201916000
H	1.987841000	-3.646061000	4.977500000
H	2.659934000	-2.046662000	4.555312000
H	2.517219000	-3.290611000	3.307891000
C	-2.757939000	-3.208521000	5.358803000
H	-3.493013000	-3.815642000	4.814492000
H	-3.327187000	-2.481567000	5.952313000
H	-2.197525000	-3.859734000	6.034451000
C	-3.704213000	-1.338176000	3.562198000
C	-4.234003000	-0.265887000	4.290340000
C	-4.568969000	-2.151064000	2.817749000
C	-5.607519000	-0.014856000	4.278967000
C	-5.942475000	-1.903349000	2.814668000
C	-6.468815000	-0.833265000	3.543798000
H	-3.561998000	0.359808000	4.876667000
H	-4.158638000	-2.978294000	2.238899000
H	-6.005601000	0.820812000	4.854105000
H	-6.602418000	-2.546367000	2.233440000
H	-7.540689000	-0.638371000	3.537615000
C	0.318891000	0.000511000	0.461788000
H	1.299583000	-0.432825000	0.697579000
H	0.376524000	1.095109000	0.470730000
H	-0.006700000	-0.318674000	-0.535163000

I	-3.026024000	0.139327000	0.330148000

15. Acyl 13a

Rh	2.623137000	-0.981214000	-0.229979000
P	3.882496000	0.776840000	0.664416000
C	0.830874000	1.430071000	-3.350449000
C	1.219436000	0.185044000	-2.592221000
C	0.676669000	-1.041324000	-2.952864000
C	1.094962000	-2.324384000	-2.520551000
C	0.591507000	-3.484591000	-3.354674000
C	0.977229000	-0.730800000	0.827376000
C	4.135472000	1.111792000	2.459572000
C	3.307937000	0.520433000	3.423015000
C	3.452742000	0.849687000	4.773045000
C	4.428002000	1.766155000	5.171691000
C	5.256516000	2.361081000	4.214620000
C	5.105758000	2.042978000	2.864523000
C	3.475725000	2.471028000	0.031937000
C	4.473208000	3.345794000	-0.422381000
C	4.144469000	4.639224000	-0.835856000
C	2.817238000	5.072611000	-0.799185000
C	1.819670000	4.206419000	-0.341892000
C	2.144091000	2.913163000	0.070647000
C	5.547351000	0.451252000	-0.044094000
C	6.640734000	0.041732000	0.729996000
C	7.847898000	-0.298180000	0.114923000
C	7.970386000	-0.244391000	-1.275761000
C	6.877926000	0.147928000	-2.055361000
C	5.671370000	0.489056000	-1.444860000
O	2.096659000	0.390174000	-1.658477000
N	1.904590000	-2.540816000	-1.485486000
O	0.672462000	0.337868000	1.323408000
H	2.554200000	-0.201266000	3.119711000
H	2.801305000	0.385721000	5.512329000
H	4.540969000	2.020720000	6.225026000
H	6.016979000	3.079749000	4.516956000
H	5.746180000	2.525096000	2.127503000
H	1.364880000	2.239411000	0.418663000
H	0.781459000	4.536619000	-0.309956000
H	2.561374000	6.080773000	-1.124810000
H	4.931050000	5.306270000	-1.186819000
H	5.512727000	3.024332000	-0.461664000

H	6.551397000	-0.017817000	1.813204000
H	8.692522000	-0.611343000	0.727172000
H	8.913338000	-0.512189000	-1.751901000
H	6.963971000	0.187470000	-3.140541000
H	4.820064000	0.790089000	-2.055584000
H	1.722191000	1.868072000	-3.819338000
H	0.086956000	1.218035000	-4.123838000
H	0.434794000	2.179708000	-2.653209000
H	1.431459000	-4.043223000	-3.788600000
H	0.022304000	-4.195789000	-2.743259000
H	-0.046462000	-3.123317000	-4.165135000
H	-0.040252000	-1.025356000	-3.769723000
C	0.081588000	-1.949920000	0.954924000
H	0.659088000	-2.789968000	1.356068000
H	-0.760701000	-1.707137000	1.612263000
H	-0.285926000	-2.236337000	-0.036879000
I	3.740951000	-2.636228000	1.615068000
C	2.383204000	-3.886000000	-1.306169000
C	1.620108000	-4.852346000	-0.642436000
C	3.639801000	-4.233646000	-1.814102000
C	2.107604000	-6.152052000	-0.494322000
C	4.123735000	-5.533856000	-1.668537000
C	3.359678000	-6.499258000	-1.007486000
H	0.642300000	-4.588011000	-0.243886000
H	4.235585000	-3.474708000	-2.320771000
H	1.503526000	-6.895510000	0.024423000
H	5.102587000	-5.791396000	-2.071552000
H	3.737960000	-7.514168000	-0.891955000

16. Acyl 14a

Rh	2.728784000	-0.698210000	-0.128656000
P	3.916683000	0.575289000	1.421288000
C	-0.042369000	2.030910000	-2.274480000
C	0.574126000	0.826422000	-1.599098000
C	-0.194346000	-0.360897000	-1.579802000
C	0.387002000	-1.626845000	-1.685297000
C	-0.445829000	-2.787548000	-2.169418000
C	1.307058000	-1.011304000	1.217730000
C	3.793248000	0.011322000	3.188018000
C	3.931203000	-1.349310000	3.505976000
C	3.853505000	-1.781889000	4.830501000
C	3.636057000	-0.862685000	5.860097000

C	3.507129000	0.493318000	5.554747000
C	3.586112000	0.928945000	4.229384000
C	3.442092000	2.359901000	1.567410000
C	4.406767000	3.377089000	1.562029000
C	4.025316000	4.714146000	1.699383000
C	2.678569000	5.049661000	1.846996000
C	1.711956000	4.039951000	1.856831000
C	2.087548000	2.703610000	1.714528000
C	5.733816000	0.640434000	1.116231000
C	6.663666000	0.609028000	2.164697000
C	8.032272000	0.678537000	1.893088000
C	8.483137000	0.785726000	0.575206000
C	7.559566000	0.822623000	-0.473677000
C	6.192846000	0.744983000	-0.205220000
N	1.826938000	0.876561000	-1.151671000
O	1.635206000	-1.909142000	-1.465672000
O	0.740681000	-0.122370000	1.819563000
H	4.105771000	-2.076589000	2.713453000
H	3.960424000	-2.842696000	5.055048000
H	3.567303000	-1.201410000	6.893493000
H	3.341530000	1.221239000	6.347912000
H	3.483490000	1.990790000	4.013013000
H	1.333276000	1.920832000	1.716731000
H	0.658470000	4.292883000	1.972593000
H	2.382962000	6.093110000	1.954307000
H	4.786873000	5.492847000	1.690848000
H	5.461241000	3.130982000	1.448599000
H	6.323253000	0.527366000	3.195922000
H	8.745149000	0.646207000	2.715851000
H	9.551198000	0.835725000	0.365161000
H	7.903498000	0.903407000	-1.504441000
H	5.481799000	0.765813000	-1.030618000
H	0.376407000	2.164331000	-3.282449000
H	-1.122037000	1.888276000	-2.375222000
H	0.150114000	2.952353000	-1.714329000
H	-0.011232000	-3.187570000	-3.095340000
H	-0.420817000	-3.596821000	-1.427624000
H	-1.483905000	-2.497934000	-2.357298000
H	-1.251634000	-0.276869000	-1.820393000
C	0.985075000	-2.477045000	1.464893000
H	1.184036000	-3.099077000	0.591225000
H	1.643133000	-2.814563000	2.277033000
H	-0.057056000	-2.557300000	1.798152000
I	4.375989000	-2.862296000	0.114204000

C	2.763722000	1.734805000	-1.828870000
C	2.908248000	3.100868000	-1.566421000
C	3.567442000	1.138251000	-2.818206000
C	3.843884000	3.853530000	-2.281041000
C	4.507655000	1.892412000	-3.520077000
C	4.649203000	3.256157000	-3.252488000
H	2.292709000	3.575142000	-0.805225000
H	3.434760000	0.078194000	-3.038368000
H	3.943390000	4.917510000	-2.069426000
H	5.122089000	1.412926000	-4.280604000
H	5.379137000	3.850157000	-3.800851000

17. Acyl 15a

Rh	2.784384000	-1.063043000	-0.201886000
P	3.816277000	0.832163000	0.673499000
C	2.272611000	0.881117000	-4.185836000
C	2.219650000	-0.179030000	-3.109146000
C	1.660107000	-1.424786000	-3.407282000
C	1.607113000	-2.586086000	-2.601977000
C	1.027829000	-3.813986000	-3.277487000
C	3.552366000	-2.218503000	1.184841000
C	4.245137000	0.901204000	2.464406000
C	3.211126000	0.669713000	3.388038000
C	3.460259000	0.741110000	4.758075000
C	4.743740000	1.044040000	5.223465000
C	5.772670000	1.285560000	4.310874000
C	5.526229000	1.218738000	2.937108000
C	3.007482000	2.471213000	0.400535000
C	3.586409000	3.618552000	0.967778000
C	3.008155000	4.872284000	0.766313000
C	1.841983000	4.991445000	0.003705000
C	1.260510000	3.853137000	-0.557871000
C	1.840442000	2.596883000	-0.364018000
C	5.416233000	0.973176000	-0.225956000
C	6.450338000	0.056212000	0.033811000
C	7.637247000	0.107379000	-0.697678000
C	7.799163000	1.057414000	-1.710667000
C	6.766234000	1.955943000	-1.989692000
C	5.580137000	1.914196000	-1.254109000
O	2.722225000	0.165505000	-1.978062000
N	2.042043000	-2.679946000	-1.341921000
O	4.680240000	-2.504177000	0.784414000

H	2.207483000	0.433130000	3.030721000
H	2.650960000	0.556072000	5.462959000
H	4.939721000	1.093435000	6.294108000
H	6.773563000	1.529254000	4.664404000
H	6.337071000	1.417354000	2.238470000
H	1.392546000	1.713693000	-0.809841000
H	0.350304000	3.938664000	-1.150323000
H	1.386341000	5.969925000	-0.147318000
H	3.466735000	5.754278000	1.210798000
H	4.491243000	3.537148000	1.569259000
H	6.325651000	-0.706184000	0.799360000
H	8.433911000	-0.602520000	-0.479296000
H	8.725109000	1.092803000	-2.284178000
H	6.880249000	2.693461000	-2.783400000
H	4.778152000	2.613286000	-1.484279000
H	3.318565000	1.161107000	-4.368738000
H	1.820175000	0.547387000	-5.124435000
H	1.755581000	1.784057000	-3.834888000
H	1.747219000	-4.643321000	-3.269314000
H	0.132244000	-4.165911000	-2.749194000
H	0.760090000	-3.589825000	-4.313114000
H	1.267591000	-1.541182000	-4.414642000
C	2.979099000	-2.771449000	2.459931000
H	3.763165000	-3.242346000	3.064694000
H	2.458004000	-1.990344000	3.021786000
H	2.225131000	-3.522554000	2.179298000
I	0.398289000	-0.654479000	0.918046000
C	1.929920000	-3.958230000	-0.697875000
C	0.744360000	-4.344825000	-0.056877000
C	3.024523000	-4.833579000	-0.704409000
C	0.658696000	-5.590546000	0.567623000
C	2.932253000	-6.080509000	-0.082501000
C	1.750511000	-6.463856000	0.556687000
H	-0.101030000	-3.658893000	-0.045899000
H	3.944251000	-4.528615000	-1.201673000
H	-0.267736000	-5.879576000	1.062569000
H	3.788950000	-6.753356000	-0.099851000
H	1.679851000	-7.437084000	1.041004000

18. Acyl 16a

Rh	2.472719000	-0.656725000	0.064312000
P	3.961200000	0.844106000	0.988931000

C	2.860940000	-0.639391000	-4.482130000
C	2.584058000	-1.010478000	-3.039210000
C	2.252116000	-2.373599000	-2.811938000
C	1.564734000	-2.885981000	-1.717731000
C	0.991437000	-4.281976000	-1.790216000
C	2.884197000	-1.999730000	1.445073000
C	4.460768000	0.376968000	2.706045000
C	3.506602000	0.517620000	3.728973000
C	3.823996000	0.168015000	5.040705000
C	5.094974000	-0.330472000	5.346679000
C	6.045562000	-0.473680000	4.334387000
C	5.731891000	-0.121726000	3.018057000
C	3.600880000	2.638595000	1.206996000
C	4.370945000	3.402314000	2.101458000
C	4.122525000	4.766115000	2.256788000
C	3.098178000	5.379444000	1.528211000
C	2.329151000	4.623843000	0.641170000
C	2.578591000	3.259004000	0.479673000
C	5.540052000	0.791132000	0.028032000
C	5.980738000	-0.447629000	-0.468412000
C	7.145704000	-0.528248000	-1.232609000
C	7.881548000	0.625299000	-1.515065000
C	7.450151000	1.859462000	-1.023008000
C	6.285802000	1.944474000	-0.257089000
N	2.598432000	-0.084662000	-2.089286000
O	1.306017000	-2.268217000	-0.606109000
O	3.917440000	-2.543060000	1.073933000
H	2.510946000	0.897636000	3.495130000
H	3.076796000	0.282815000	5.825057000
H	5.341679000	-0.606581000	6.371507000
H	7.037748000	-0.859839000	4.564298000
H	6.483924000	-0.234771000	2.238317000
H	1.967352000	2.675494000	-0.205126000
H	1.527350000	5.093306000	0.072406000
H	2.899225000	6.443223000	1.656751000
H	4.726008000	5.346961000	2.953160000
H	5.164307000	2.932470000	2.681481000
H	5.413330000	-1.351612000	-0.254386000
H	7.475349000	-1.495268000	-1.610189000
H	8.787328000	0.562710000	-2.117613000
H	8.016557000	2.764061000	-1.240497000
H	5.952724000	2.915322000	0.104831000
H	3.775447000	-0.041070000	-4.571745000
H	2.954809000	-1.537128000	-5.098902000

H	2.040100000	-0.028687000	-4.884596000
H	1.389994000	-4.887284000	-0.965324000
H	-0.098418000	-4.237783000	-1.662394000
H	1.220416000	-4.769788000	-2.742418000
H	2.361140000	-3.040134000	-3.663860000
C	2.012915000	-2.526109000	2.543809000
H	2.511231000	-3.342439000	3.079257000
H	1.722258000	-1.719013000	3.224194000
H	1.093455000	-2.890000000	2.064400000
I	0.314498000	0.364435000	1.244155000
C	2.610465000	1.283352000	-2.501642000
C	1.382686000	1.953907000	-2.631754000
C	3.797352000	1.977891000	-2.765260000
C	1.345931000	3.292549000	-3.023338000
C	3.755505000	3.319371000	-3.151848000
C	2.533243000	3.982789000	-3.284333000
H	0.461263000	1.413480000	-2.415461000
H	4.751444000	1.461236000	-2.667513000
H	0.385126000	3.797301000	-3.120208000
H	4.687218000	3.848020000	-3.352436000
H	2.507175000	5.029680000	-3.584631000

19. Acyl 18a

Rh	2.549714000	-0.950013000	-0.199501000
P	0.639775000	-1.221656000	0.962078000
C	0.949566000	0.387894000	-4.145430000
C	1.517527000	-0.480127000	-3.040238000
C	1.638331000	-1.853518000	-3.354868000
C	2.294275000	-2.852655000	-2.630237000
C	2.445291000	-4.225735000	-3.241762000
C	3.267944000	0.669414000	0.670542000
C	0.143024000	-2.995236000	1.179027000
C	0.869835000	-4.052026000	0.619477000
C	0.405864000	-5.365821000	0.743509000
C	-0.777827000	-5.634107000	1.431483000
C	-1.507456000	-4.580967000	1.993516000
C	-1.056223000	-3.268615000	1.860917000
C	-0.900491000	-0.601004000	0.136630000
C	-1.853502000	0.212359000	0.766683000
C	-3.019106000	0.586393000	0.091926000
C	-3.251546000	0.146427000	-1.211543000

C	-2.317618000	-0.686757000	-1.836592000
C	-1.154081000	-1.061315000	-1.166840000
C	0.591767000	-0.513280000	2.650467000
C	0.759724000	-1.335147000	3.776336000
C	0.767771000	-0.782204000	5.056866000
C	0.617129000	0.597291000	5.227122000
C	0.472965000	1.423393000	4.110162000
C	0.473369000	0.874659000	2.827452000
N	1.857100000	0.083812000	-1.877127000
O	2.811642000	-2.708439000	-1.460342000
O	2.903876000	1.498989000	1.472757000
H	1.792856000	-3.850186000	0.079517000
H	0.977723000	-6.180506000	0.300252000
H	-1.135600000	-6.658741000	1.528739000
H	-2.433193000	-4.779206000	2.532115000
H	-1.641129000	-2.456817000	2.292156000
H	-0.440107000	-1.718455000	-1.663174000
H	-2.498274000	-1.050267000	-2.847668000
H	-4.160392000	0.440791000	-1.735649000
H	-3.747723000	1.219951000	0.595746000
H	-1.702134000	0.554006000	1.787730000
H	0.887048000	-2.409056000	3.653694000
H	0.895168000	-1.432290000	5.921209000
H	0.621342000	1.027821000	6.228119000
H	0.371713000	2.500533000	4.234019000
H	0.393970000	1.532987000	1.966963000
H	1.743552000	0.964711000	-4.638159000
H	0.467449000	-0.237925000	-4.901648000
H	0.222097000	1.108224000	-3.752373000
H	3.510649000	-4.484685000	-3.298140000
H	1.969044000	-4.969124000	-2.588213000
H	2.004673000	-4.290181000	-4.241104000
H	1.252950000	-2.143436000	-4.329508000
C	4.678196000	0.711129000	0.043197000
H	4.854916000	-0.016164000	-0.762666000
H	4.847769000	1.721499000	-0.346826000
H	5.386956000	0.488607000	0.849883000
I	3.905517000	-2.366770000	1.710586000
C	1.845482000	1.521509000	-1.806234000
C	0.816431000	2.214275000	-1.158651000
C	2.898712000	2.242694000	-2.386124000
C	0.844267000	3.607380000	-1.087994000
C	2.922239000	3.636838000	-2.315679000
C	1.896031000	4.325543000	-1.663959000

H	-0.008414000	1.656180000	-0.719630000
H	3.699662000	1.703468000	-2.892060000
H	0.035858000	4.134469000	-0.582187000
H	3.747420000	4.183494000	-2.770194000
H	1.914887000	5.413094000	-1.606097000
